# Application of Vinamidinium Salt Chemistry for a Palladium Free Synthesis of Anti-Malarial MMV048: A “Bottom-Up” Approach

**DOI:** 10.1021/acs.orglett.1c01725

**Published:** 2021-06-29

**Authors:** Dinesh J. Paymode, Le Chang, Dan Chen, Binglin Wang, Komirishetty Kashinath, Vijayagopal Gopalsamuthiram, D. Tyler McQuade, N. Vasudevan, Saeed Ahmad, David R. Snead

**Affiliations:** † Medicines for All Institute, 737 North Fifth Street, Box 980100, Richmond, Virginia 23298, United States; § WuXi AppTec (Wuhan) Co. Ltd., Wuhan East Lake High-tech Development Zone, Wuhan 430075, P. R. of China

## Abstract

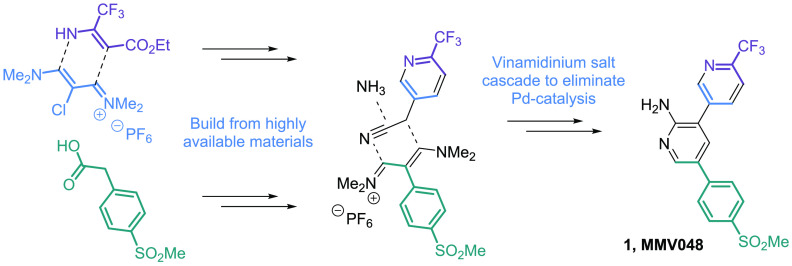

MMV390048 (**1**) is a clinical compound under investigation for antimalarial activity. A new synthetic route was developed which couples two aromatic fragments while forming the central pyridine ring over two steps. This sequence takes advantage of raw materials used in the existing etoricoxib supply chain and eliminates the need for palladium catalysts, which were projected to be major cost-drivers.

MMV390048 (**1**) is an emerging clinical candidate under development by Medicines for Malaria Venture (MMV) in Phase I trials (NCT02230579, NCT02281344, and NCT02554799) and shows potential to be used as a single dose cure for malaria.^1^ New malaria treatments with novel mechanism of action are needed as drug resistance develops for the artemesinins and chloroquine. Development of an economical supply route is an important goal because antimalarial treatments face high downward cost pressures.

Retrosynthesis is a tool for what can be viewed as a top down approach toward synthetic design. One examines the final target seeking logical bond disconnects, and then deconstructs the molecule bond-by-bond until reaching seemingly simple starting materials which one might presume to be at the base of the chemical supply chain. A retrosynthetic analysis of MMV390048 points toward assembly of the triaromatic core through a series of cross-coupling reactions mediated by palladium catalysis (Figure 1). This is a very logical and efficient sequence of bond disconnects, and it excels for a given policy which seeks to provide access to a diverse range of biologically active structures. All syntheses to date have relied upon this approach.^1a,2^

**Figure 1:**
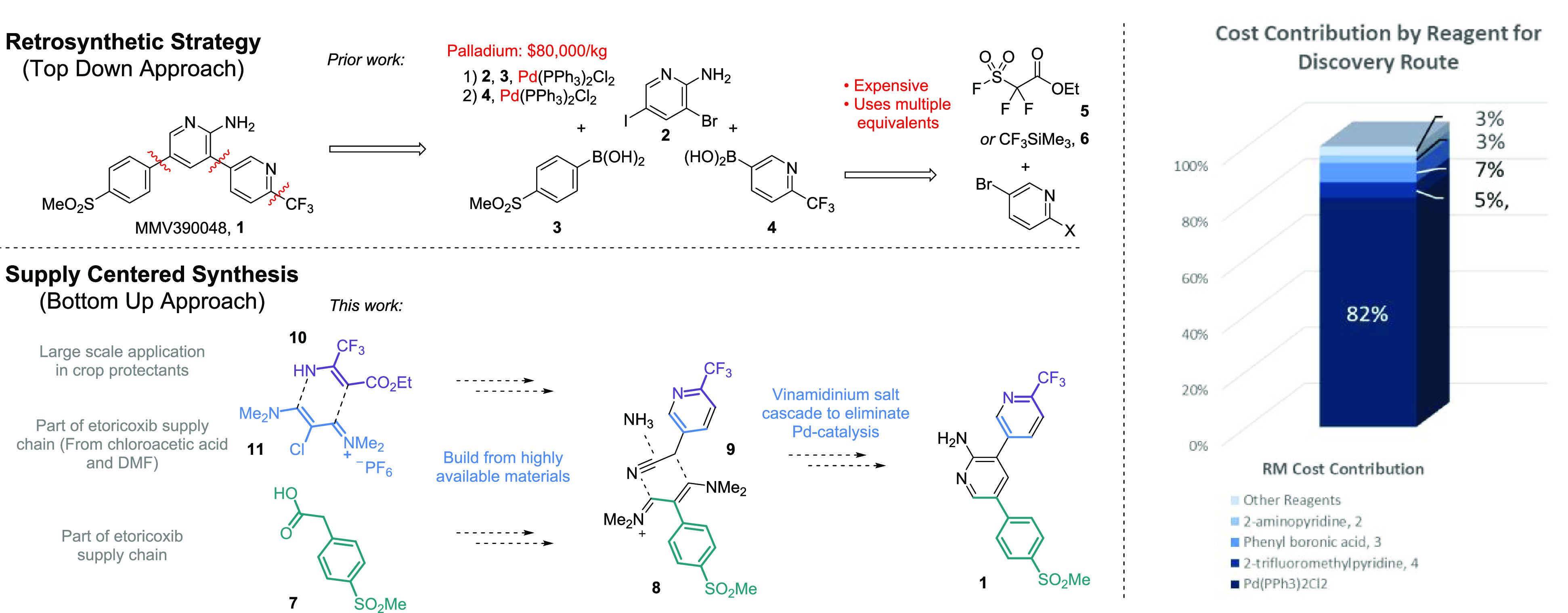
Adopting a bottom-up synthetic approach can solve cost issues related to MMV390048.

However, for a given policy which seeks to minimize cost and production time as well as improve sustainability, this route might not be optimal.^3^ Modeling suggests that the predominant cost driver is the use of palladium catalysts and that cost is sensitive to the equivalents of expensive reagents used for trifluoromethylation. Palladium metal itself is a particular problem because its cost ($80,000/kg) has risen dramatically over the past decade due to increased demand in catalytic converters. Also, the starting materials of this route are not abundantly available and require custom synthesis themselves, as they are fine chemicals without independent market application. This increases API production cycle time and also consumption of solvents and reagents in route to making these starting materials.

Perhaps some of these drawbacks could be addressed by instead adopting a bottom-up approach, where the emphasis of route design is placed on the starting materials rather than final product. It is referred to as a bottom-up approach since the ideal materials are those which are positioned at the base of the chemical supply chain due to their independent market consumption and thus abundant availability. Inventing from the pool of available materials can have large benefits for a given policy which emphasizes cost, shortened production time, and sustainability. By adopting supply centered synthetic analysis as a tool, we hoped to select materials which would negate the need for palladium (cross-coupling) and expensive trifluoromethylating reagents (Figure 1).

From this perspective, phenyl acetic acid derivative **7** presents some intrigue. It is part of the etoricoxib (an API used to treat rheumatoid arthritis) supply chain^4^ and already consumed in multimetric ton quantities with the price <$50/kg (Figure 2). The acetic acid unit located on the sulfonyl benzene scaffold presents a handle for further functionalization with a goal of constructing the central pyridine ring in a *de novo* manner. Acetic acid derivatives can be converted into vinamidinium salts, which in turn can be used to form pyridine rings.^5^ Reaction of the electrophilic iminium salt **8** with a carbon centered nucleophile such as nitrile **9** as depicted in Figure 1 would eliminate the need for palladium cross-couplings altogether—the biaryl bonds are already present in the starting material.

**Figure 2:**
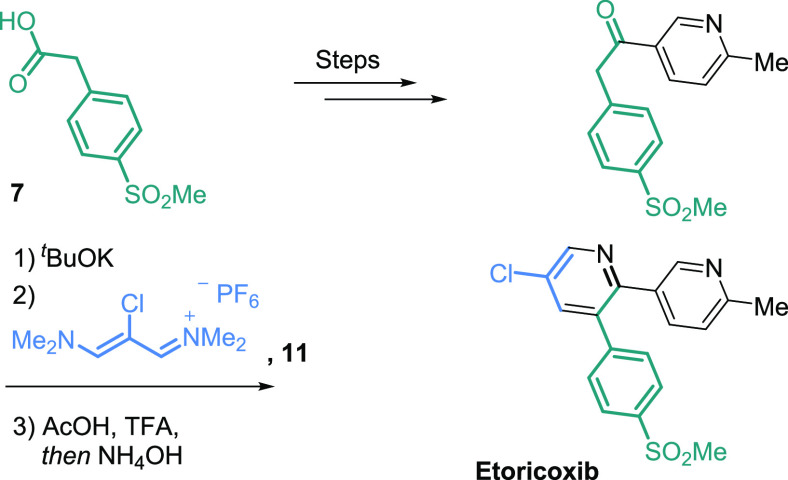
“Recycling” Merck’s etoricoxib supply chain.

Possibly challenges associated with the trifluoromethyl group’s installation could also be solved through supply centered synthesis. Ethyl 4,4,4-trifluoromethyl-3-aminocrotonate **10** is available in large quantities and inexpensive. It is used in production of crop protectants^6^ and is in the same value chain as ethyl acetate, trifluoroacetic acid, and ammonia.^7^ The parent trifluoroacetoacetate reagent reacts with electrophilic C_3_ synthons to form pyridine rings which could be useful in subsequent downstream chemistry.^8^ Perhaps vinamidinium salt **11**, which is made from chloroacetic acid and also part of the supply route to etoricoxib,^4^ could be used as that C_3_ fragment. A retrosynthetic approach might not suggest selection of the aminocrotonate reagent due to the resultant ester substitution pattern of the pyridine; however, its economical nature warrants consideration of how this starting material could be synthetically fit for purpose. With these objectives in mind, we set out to improve the route to MMV390048.

We commenced our investigation by looking toward economical construction of 5-bromo-2-trifluoromethylpyridine **12** en route to the nitrile **9**. The straightforward bond-disconnect which makes use of 5-bromo-2-halopyridine was examined first. Significant literature precedent exists for this transformation; however, conditions tend to favor selection of methyl difluoro(fluorosulfonyl)acetate,^9^ CF_3_Si(Me)_3_,^10^ CF_3_I,^11^ chlorodifluoroacetate,^12^ and their synthons, all of which are of considerable expense (∼$150/kg and above). Unfortunately, an excess of reagent is frequently necessary. We hoped to find conditions which would mitigate these factors.

Reaction optimization was explored extensively, and the major findings are presented in Table 1. In all cases, alkylation of the 2-iodo-5-bromopyridine proceeded in substantially higher yield than 2,5-dibromopyridine (judged by liquid chromatography area percent, LCAP). Notably, selection of the aryl iodide reduced the loading of methyl difluoro(fluorosulfonyl)acetate by 70% and significantly increased the yield of the trifluoromethylated product (entries 1 and 2), very important considerations given the high cost of the reagent. Ruppert’s reagent (CF_3_SiMe_3_) can be employed as a viable alternative (entries 3 and 4); however, higher consumption of the trifluoromethylating reagent is observed (3 equiv), and despite being made from simple starting materials, this reagent is of similar price to the sulfonyl fluoride. Low cost trifluoromethylation reagents were explored with high anticipation, but reactivity did not meet desired expectations (entries 10 and 11). Nevertheless, the results from entry 2 provided acceptable results and were scaled up to 10 g, and 5-bromo-2-trifluoromethylpyridine **12** was isolated in 78% yield. The 5-bromopyridine was easily converted to the nitrile in 67% over two steps (Figure 3, 85% and 79% respectively). **12** was treated with *tert*-butyl cyanoacetate in basic media followed by sodium chloride promoted decarboxylation to give **9**.

**Table 1:**
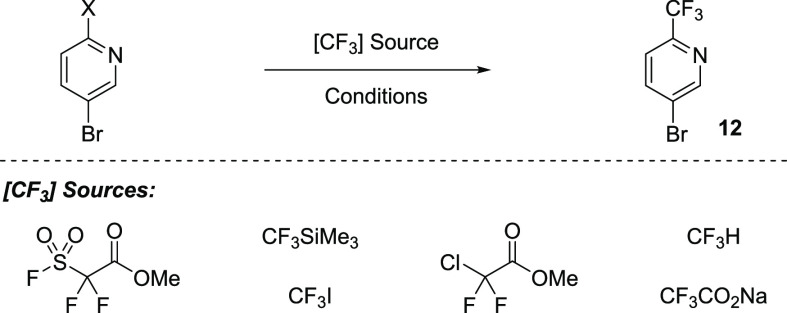
Optimizing Trifluoromethylation of Pyridine

Entry	X =	[CF_3_] (equiv)	Solvent	Temp (°C)	Time (h)	Yield, **12** (LCAP)
1^a^	Br	–SO_2_F (5)	DMF	100	16	66%
2^a^	I	–SO_2_F (1.5)	NMP	80	16	97%
3^b^	Br	CF_3_SiMe_3_ (3)	DMSO	60	20	40%
4^b^	I	CF_3_SiMe_3_ (3)	DMSO	60	16	95%
5^b^	I	CF_3_SiMe_3_ (2)	DMSO	60	16	70%
6^b^	I	CF_3_SiMe_3_ (1)	DMSO	60	16	40%
7^c^	Br	CF_3_I (5)	DMF	120	16	41%
8^c^	I	CF_3_I (3)	DMF	120	16	71%
9^d^	I	–CF_2_Cl (3)	DMF	120	16	56%
10^e^	I	CF_3_H (6)	DMF	50	16	0%
11^f^	I	CF_3_CO_2_Na (2)	NMP	150	16	0%

a Cul (1.5 equiv).

b KF (3 equiv), B(OMe)_3_ (3 equiv), Cul (0.2 equiv), 1,10-phenanthroline (0.2 equiv).

c Cu (2 equiv), reaction run in sealed tube.

d Cul (2 equiv), KF (3 equiv).

e CuCl (3 equiv), *t*BuOK (3 equiv), 1,10-phenanthroline (0.2 equiv), reaction run in sealed tube.

f Cul (2 equiv).

**Figure 3:**
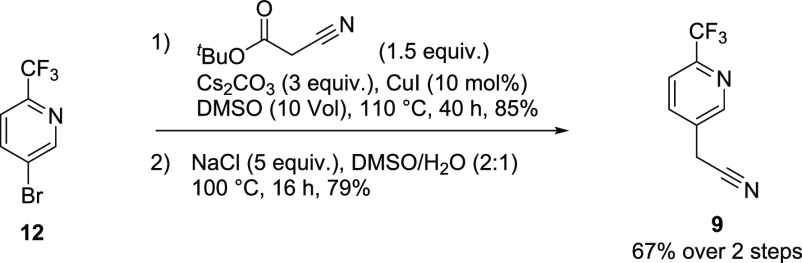
Conversion of 5-bromo-2-trifluoromethylpyridine to nitrile **9**.

Perhaps the pyridine ring could instead be built from ethyl 4,4,4-trifluoromethyl-3-aminocrotonate **10**, by reacting it with **11** which is made from chloroacetic acid, DMF, POCl_3_, and hexafluorophosphoric acid (Figure 4).^4^ The aminocrotonate is part of the very inexpensive ethyl trifluoroacetate supply chain (<$10/kg) which would be beneficial in the replacement of methyl difluoro(fluorosulfonyl)acetate. Initial attempts at this reaction focused on use of ethyl trifluoroacetoacetate in conjunction with ammonia, but this concept worked much better when using the preformed enamine **10** to give the ethyl ester analogue of the desired compound **14**. Decarboxylation was affected by reacting **13** with LiCl at high temperature giving 5-chloro-2-trifluoromethylpyridine in 62% yield (47% over two steps) from quite inexpensive materials. The chloropyridine was then converted to cyanoacetate **15** under slightly modified conditions notably replacing cesium carbonate with potassium carbonate, and **15** was decarboxylated in good yield (79%) to reach **9**. The step count for production of intermediate **9** is one step shorter by the vinamidinium route as 5-bromo-2-iodopyridine is made in two steps from 2-aminopyridine.^13^

**Figure 4:**
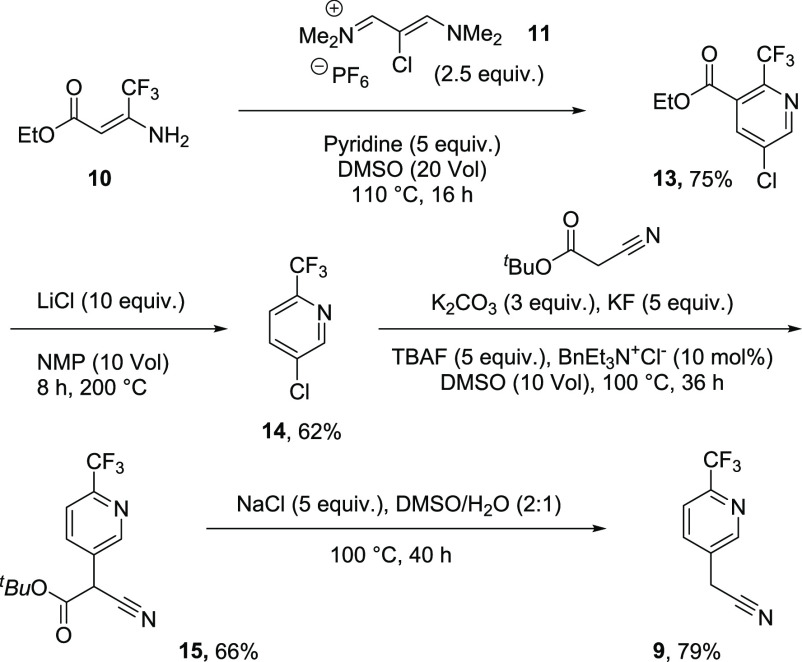
Synthesis of **9** from highly available aminocrotonate **10**.

We concluded our investigation by testing the key tenet: that the two terminal aromatic subunits could be coupled with simultaneous construction of the central aminopyridine ring and without need for palladium catalysis. In order to probe the validity of the hypothesis, the vinamidinium salt of **7** was made by reacting POCl_3_ and DMF with the sulfonylphenyl acetic acid (Figure 5). Exchanging the chloride anion with a hexafluorophosphate counterion afforded an easily isolable solid in very high yield (95%). With this key intermediate in hand, **8** and **9** were coupled using KO^*t*^Bu to form penultimate intermediate **16** in very good yield (89%). Proof-of-concept for the synthesis was established by simple reaction of **16** with ammonia, generating MMV390048 in good yield (83%). Notably, the final product precipitated from solution and was easily isolated by filtration. This constitutes a new bond-forming strategy to reach MMV390048 in a six-step longest linear sequence at gram scale.

**Figure 5:**
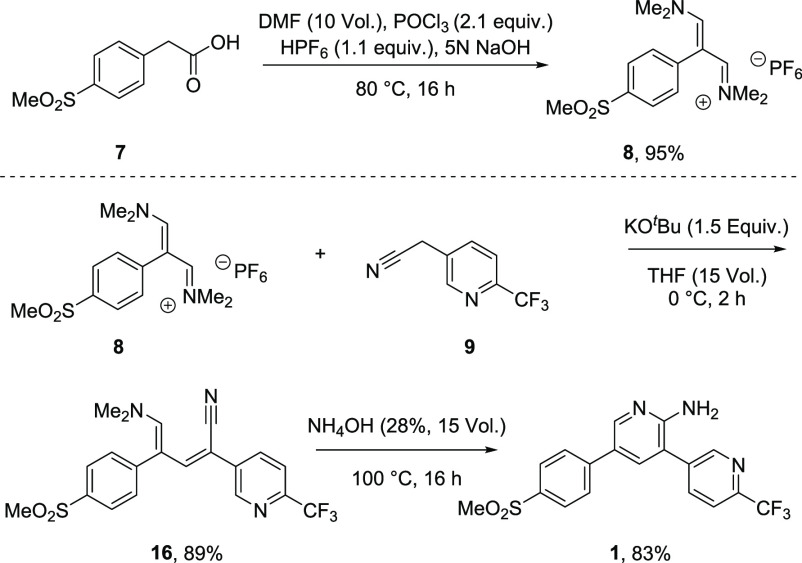
A metal-free coupling of vinamidinium salt **8** and nitrile **9** which eliminates the need for palladium metal.

In conclusion, a new palladium-free route has been developed for MMV390048 to eliminate the need for costly metal catalysts and expensive trifluoromethylating reagents. Designing syntheses from the bottom-up and selecting from the pool of highly available materials constitute an essential strategy in producing cost-effective solutions to the above challenges. As a result, the raw material costs associated with this antimalarial drug candidate were reduced 90%, and a concise synthesis was completed in a longest linear sequence of six steps. There is potential to reduce the step count and improve yields further through process optimization.

## Supporting Information Available

Optimization tables, experimental procedures and data, and NMR spectra (PDF)
