# Supplementary material for: Application of Vinamidinium Salt Chemistry for a Palladium Free Synthesis of Anti-Malarial MMV048: A “Bottom-Up” Approach
Source: Org Lett. 2021 Jun 29;23(14):5400–4. doi: 10.1021/acs.orglett.1c01725 (PMC9385109; doi:10.1021/acs.orglett.1c01725)
Supplement: Supplementary file 1 — ol1c01725_si_001.pdf [file ol1c01725_si_001.pdf]

Application of Vinamidinium Salt Chemistry for a Palladium Free Synthesis of Anti-Malarial MMV048: A "Bottom-Up" Approach

[†]Dinesh J. Paymode, [§]Le Chang, [§]Dan Chen, [§]Binglin Wang, [†]Komirishetty Kashinath, [†]Vijayagopal Gopalsamuthiram, [†]D. Tyler McQuade, [†]N. Vasudevan, [†]Saeed Ahmad, and [†]David R. Snead*

Medicines for All Institute, 737 N 5th St., Box 980100, Richmond, Virginia 23298

Supplemental Information:

Table of Contents:

General Remarks 2
Optimization Tables 3
Experimental Procedures and Data 7
References 12
^{1}H, ^{13}C and ^{19}F NMR Spectra 13
HPLC Chromatogram of MMV0048..24

General Remarks:

Instrumentation: For all compounds, ^1H and ^{13}C NMR spectra were recorded on a Bruker Avance III 600 MHz spectrometer. Chemical shifts were measured relative to the residual solvent resonance for ^1H and ^{13}C NMR ($CDCl_3$ = 7.26 ppm for ^1H and 77.0 ppm for ^{13}C, DMSO-d_6 = 2.50 ppm for ^1H and 39.5 ppm for ^{13}C, CD_3CN = 1.94 ppm for ^1H and 1.3, 118.3 ppm for ^{13}C). Coupling constants *J* are reported in hertz (Hz). The following abbreviations were used to designate signal multiplicity: s, singlet; d, doublet; t, triplet; q, quartet, p, pentet; dd, doublet of doublet; ddd, doublet of doublet of doublet; dt, double of triplet; ddt, doublet of doublet of triplet; m, multiplet; br, broad. Reactions were monitored by HPLC using the methods indicated. Mass spectroscopy was carried out on a Perkin Elmer Axion 2 ToF mass spectrometer, in positive ion mode, with cylinder voltage of -3.5 kV, endplate voltage -5 kV, capillary entrance voltage -6 kV and flight tube voltage -8 kV. Glassware was oven-dried at 120 °C, assembled while hot, and cooled to ambient temperature under an inert atmosphere. Unless noted otherwise, reactions involving air sensitive reagents and/or requiring anhydrous conditions were performed under a nitrogen atmosphere. Heating metal blocks were used as a heat source for heating reaction.

Reagents and solvents. Reagents and solvents were purchased from Aldrich Chemical Company, Fisher Scientific, Alfa Aesar, Acros Organics, Oakwood, or TCI. Liquid reagents were purified by distillation when necessary. Unless otherwise noted, solid reagents were used without further purification. Methylene chloride (DCM) and dimethylformamide (DMF) taken from a solid-sorbent Solvent Dispensing System purchased from Pure Process Technologies or distilled as described in the literature.

Optimization Tables:

Table S1: Optimization for trifluoromethylation of 5-bromo-2-halopyridine.

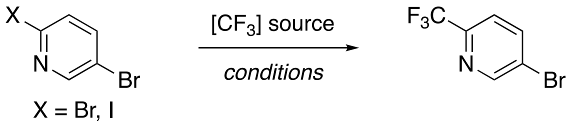

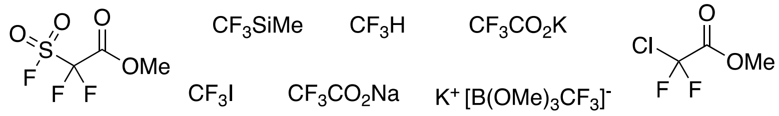

Entry	X	$[CF_3]$ source (eq.)	Reagents (eq.)	Solvent	Temp. (°C)	Time (h)	Yield (LCAP)
1.	Br	$-SO_2F$ (5)	CuI (1.5)	DMF	100	16	66
**2.**	**I**	**$-SO_2F$ (1.5)**	**CuI (1.5)**	**NMP**	**80**	**16**	**97**
3.	I	$-SO_2F$ (2.5)	CuI (2.5)	DMF	80	16	87
4.	I	$-SO_2F$ (1)	CuI (1)	DMF	80	16	86
5.	Br	CF_3SiMe_3 (3)	CuI (0.2), KF (3), $B(OMe)_3$ (3), 1,10-phenanthroline (0.2)	DMSO	60	20	40
6.	I	CF_3SiMe_3 (1.1)	CuI (1.2), KF (1.2)	NMP	50	24	66
7.	I	CF_3SiMe_3 (3)	CuI (0.2), KF (3), $B(OMe)_3$ (3), 1,10-phenanthroline (0.2)	DMSO	60	16	95
8.	I	CF_3SiMe_3 (2)	CuI (0.2), KF (3), $B(OMe)_3$ (3), 1,10-phenanthroline (0.2)	DMSO	60	16	70
9.	I	CF_3SiMe_3 (1)	CuI (0.2), KF (3), $B(OMe)_3$ (3), 1,10-phenanthroline (0.2)	DMSO	60	16	40
10.	Br	CF_3I (5)	Cu (2), in sealed tube	DMF	120	16	41
11.	I	CF_3I (3)	Cu (2), in sealed tube	DMF	120	16	71
12.	I	CF_3I (3)	Cu (2), in sealed tube	NMP	120	32	42
13.	I	$-CF_2Cl$ (3)	CuI (2), KF (3)	DMF	120	16	56
14.	I	CF_3H (6)	CuCl (3), KO^tBu (3), 1,10-phenanthroline (0.2), in sealed tube	DMF	50	16	-
15.	I	$KB(OMe)_3CF_3$ (2)	CuI (0.2), 1,10-phenanthroline (0.2)	DMSO	60	16	75
16.	I	CF_3CO_2Na (2)	CuI (2)	NMP	150	16	-
17.	I	CF_3CO_2Na (3)	Cu (0.3), Ag_2O (0.3)	NMP	150	16	37
18.	I	CF_3CO_2K (3)	Cu (0.3), Ag_2O (0.3)	NMP	150	16	25

Table S2: Optimization for synthesis of nitrile derivative from 5-bromo-2-trifluoromethylpyridine by S_NAr reaction.

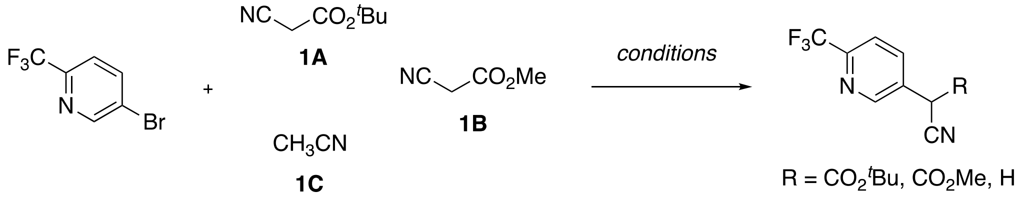

Entry	[CN] source (eq.)	Reagents (eq.)	Solvent	Temp. (°C)	Time (h)	Yield (LCAP)
1.	**1A** (2)	Cs_2CO_3 (4), CuI (0.1)	DMF	110	12	73
2.	**1A** (1.5)	KO*t*Bu (3), CuI (0.1)	DMF	110	16	50
3.	**1A** (1.5)	Cs_2CO_3 (3), TBAB (0.1)	DMSO	110	40	88
4.	**1A** (1)	KO*t*Bu (2.5), $Pd(OAc)_2$ (0.02), dppf (0.04)	1,4-dioxane	70	12	50
5.	**1B** (1.1)	KO*t*Bu (2.5), $Pd(dppf)_2Cl_2$ (0.1)	1,4-dioxane	70	16	79
6.	**1B** (2)	Cs_2CO_3 (4), CuI (0.1)	DMF	100	24	45
7.	**1B** (1.5)	Cs_2CO_3 (3), TBAB (0.1)	DMSO	110	40	88
**8.**	**1B (1.3)**	**Cs_2CO_3 (3), CuI (0.1)**	**DMSO**	**110**	**40**	**89**
9.	**1C** (2)	Cs_2CO_3 (4), CuI (0.1), in sealed tube	DMF	110	12	-
10.	**1C** (1.5)	*n*BuLi (1.5)	THF	-78 to 25	24	-
11.	**1C** (1.5)	LDA (1.5)	DMPU	0 to 25	12	-

Table S3: Optimization for cyclization reaction of trifluoroacetate derivative with vinamidinium salt.

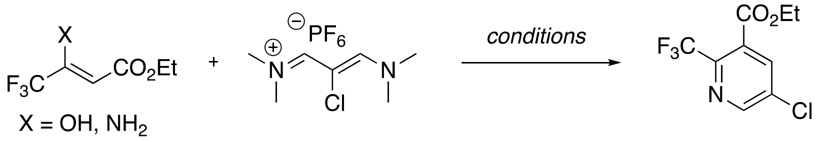

**Entry**	**X**	**Vinamidinium salt (eq.)**	**Reagents (eq.)**	**Solvent**	**Temp. (°C)**	**Time (h)**	**Yield (LCAP)**
1.	OH	1.5	KO^{t}Bu (1.1), DABCO (1.1), NH_4OAc (2)	THF	0 to 80	24	-
2.	OH	1.5	KO^{t}Bu (1.6), TFA (0.8), AcOH (7), NH_4OH (15)	THF	0 to 90	24	-
3.	OH	2.5	-	pyridine	100	24	-
4.	NH_2	1.5	Cs_2CO_3 (2), open vial	DMF	60	16	31
5.	NH_2	1.5	K_2CO_3 (2), open vial	DMF	60	16	22
6.	NH_2	1.5	pyridine (5), open vial	DMSO	110	8	66
7.	NH_2	2.5	DIPEA (5), open vial	DMSO	110	16	45
8.	NH_2	2.5	TEA (7), open vial	DMSO	100	16	31
**9.**	**NH_2**	**2.5**	**pyridine (5), open vial**	**DMSO**	**110**	**16**	**78**

Table S4: Optimization for synthesis of nitrile derivative from 5-chloro-2-trifluoromethylpyridine by S_NAr reaction.

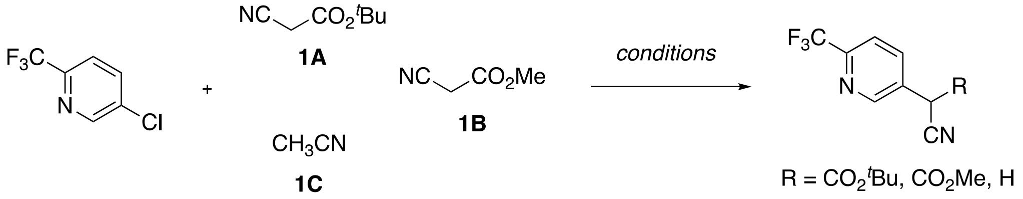

Entry	[CN] source (eq.)	Reagents (eq.)	Solvent	Temp. (°C)	Time (h)	Yield (LCAP)
1.	**1A** (1.5)	Cs_2CO_3 (3), CuI (0.1)	DMF	110	32	18
2.	**1A** (1.5)	K_2CO_3 (3), $BnEt_3NCl$ (0.1), KF (5), TBAB (5)	DMSO	100	16	56
3.	**1B** (1.5)	KO^{t}Bu (3), CsF (5), TBAB (3)	DMSO	100	16	43
4.	**1B** (1.5)	Cs_2CO_3 (3), CuI (0.1)	DMF	110	27	32
5.	**1B** (1.5)	Cs_2CO_3 (3), TBAB (0.1)	DMSO	110	16	21
6.	**1B** (1.3)	K_2CO_3 (3), $BnEt_3NCl$ (0.1), CsF (5), TBAB (3)	DMSO	100	24	63
**7.**	**1B (1.5)**	**K_2CO_3 (3), $BnEt_3NCl$ (0.1), KF (5), TBAB (5)**	**DMSO**	**100**	**36**	**78**
8.	**1C** (3)	K_2CO_3 (3), $BnEt_3NCl$ (0.1), CsF (5), TBAB (3)	DMSO	100	16	-
9.	**1C** (1.5)	NaH (3)	DMF	0 to 70	16	-

Experimental Procedures and Data:

5-Bromo-2-(trifluoromethyl)pyridine (12):

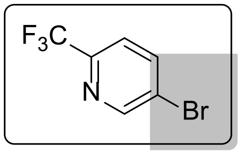

To a solution of 5-bromo-2-iodopyridine (10.0 g, 35.22 mmol, 1.0 eq.) in NMP (100 mL), $FSO_2CF_2CO_2Me$ (10.15 g, 52.84 mmol, 1.5 eq.) and CuI (10.06 g, 52.84 mmol, 1.5 eq.) were added at room temperature under argon atmosphere. The suspension was stirred for 16 hours at 80 °C. After completion of the reaction, the reaction suspension was distilled at 90 °C to 100 °C under reduced pressure to give a mixture of NMP and product. The solution was diluted with diethyl ether (200 ml), washed with water (2 X 100 mL) and brine (100 mL). The organic layer was dried over Na_2SO_4 and solvent was removed under reduced pressure at 30 °C to afford the desired product **12** as white-yellow solid (6.2 g, 78% yield).

Data matched with those previously reported.[1]

Ethyl 5-chloro-2-(trifluoromethyl)nicotinate (13):

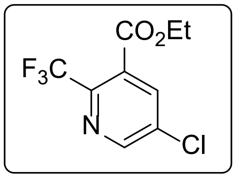

To a solution of aminocrotonate **10** (11.0 g, 60 mmol, 1.0 eq.) in DMSO (220 mL), vinamidinium salt[2] **11** (46.0 g, 150 mmol, 2.5 eq.) and pyridine (23.8 g, 300 mmol, 5.0 eq.) were added at room temperature. The suspension was stirred for 16 hours at 110 °C in an unsealed round bottom flask. The reaction mixture was cooled, diluted with brine (300 ml) and the aqueous phase was extracted with EtOAc (3 X 150 mL). The organic layer was washed with brine (3 X 100 mL), dried over Na_2SO_4 and solvent was removed under reduced pressure. The crude residue was purified by silica gel column chromatography (0% to 30% EtOAc in hexanes) to give the titled product **13** as colorless oil (12.5 g, 75% yield).

^{1}H NMR (600 MHz, Chloroform-*d*) δ 8.72 (d, J = 2.4 Hz, 1H), 8.08 (d, J = 2.4 Hz, 1H), 4.42 (q, J = 7.2 Hz, 2H), 1.39 (t, J = 7.2 Hz, 3H); **^{13}C NMR** (150 MHz, Chloroform-*d*) δ 164.1, 149.7, 143.6 (q, J = 35.9 Hz), 137.9, 134.9, 129.1, 120.8 (q, J = 274.8 Hz), 63.0, 13.8; **^{19}F NMR** (565 MHz, Chloroform-*d*) δ -64.1 ppm.

5-Chloro-2-(trifluoromethyl)pyridine (14):

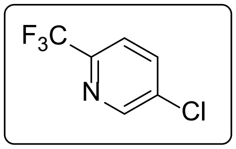

To a solution of pyridine derivative **13** (15.0 g, 54 mmol, 1.0 eq.) in NMP (150 mL), LiCl (25.0 g, 540 mmol, 10.0 eq.) was added at room temperature. The suspension was stirred for 15 hours at 200 °C. The reaction suspension was distilled at 120 °C to 140 °C under high vacuum to give a mixture of NMP and product. The solution was diluted with EtOAc (50 ml), washed with brine (4 X 30 mL). The organic layer was dried over Na_2SO_4 and solvent was removed nitrogen gas flow to afford the desired product **14** as white solid (6.1 g, 62% yield).

^{1}H NMR (600 MHz, Chloroform-*d*) δ 8.67 (d, J = 2.5 Hz, 1H), 7.84 (dd, J = 8.5, 2.4 Hz, 1H), 7.63 (d, J = 8.4 Hz, 1H); **^{13}C NMR** (150 MHz, Chloroform-*d*) δ 149.2, 146.3 (q, J = 35.5 Hz), 137.1, 135.2, 121.4 (q, J = 2.8 Hz), 121.2 (q, J = 273.9 Hz); **^{19}F NMR** (565 MHz, Chloroform-*d*) δ -67.8 ppm.

Data matched with those previously reported.[3]

***tert*-Butyl 2-cyano-2-(6-(trifluoromethyl)pyridin-3-yl)acetate (15):**

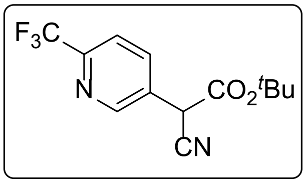

<u>*Synthesis from 5-bromo-2-trifluoromethylpyridine*:</u> To a solution of bromopyridine **12** (5.0 g, 22.12 mmol, 1.0 eq.) and *tert*-butyl-2-cyanoacetate (4.06 g, 28.76 mmol, 1.3 eq.) in DMSO (50 mL), Cs_2CO_3 (21.63 g, 66.37 mmol, 3.0 eq.) and CuI (421 mg, 2.21 mmol, 10 mol%) were added at room temperature under argon. The suspension was stirred for 40 hours at 110 °C. The reaction suspension was cooled, filtered through Celite and the filtrate was diluted ethyl acetate (150 ml), washed with water (2 X 100 mL) and brine (100 mL). The organic solution was dried over Na_2SO_4 and solvent was removed under reduced pressure. The crude residue was purified by silica gel column chromatography (20% to 60% EtOAc in hexanes) to give the titled product **15** as a brown solid (5.4 g, 85% yield).

<u>*Synthesis from 5-chloro-2-trifluoromethylpyridine*:</u> To a solution of chloropyridine **14** (1.0 g, 5.4 mmol, 1.0 eq.) and *tert*-butyl-2-cyanoacetate (1.2 g, 8.2 mmol, 1.5 eq.) in DMSO (10 mL), K_2CO_3 (2.3 g, 16.4 mmol, 3.0 eq.), $BnEt_3NCl$ (100 mg, 0.5 mmol, 0.1 eq.), KF (1.6 g, 27.3 mmol, 5.0 eq.) and TBAF (1M solution, 27.3 mL, 27.3 mmol, 5.0 eq.) were added at room temperature under argon. The suspension was stirred for 36 hours at 100 °C. The reaction suspension was cooled and filtered through Celite. The filtrate was diluted water (50 ml), extracted with EtOAc (5 X 30 mL).

The combined organic layers were washed with brine (50 mL), dried over Na_2SO_4 and solvent was removed under reduced pressure. The crude residue was purified by silica gel column chromatography (20% to 60% EtOAc in hexanes) to give the titled product **15** (1 g, 66% yield).

^{1}H NMR (600 MHz, Chloroform-*d*) δ 8.79 (d, *J* = 2.3 Hz, 1H), 8.03 (dd, *J* = 8.2, 2.3 Hz, 1H), 7.77 (d, *J* = 8.3 Hz, 1H), 4.75 (s, 1H), 1.47 (s, 9H); **^{13}C NMR** (150 MHz, Chloroform-*d*) δ 162.2, 149.4, 148.9 (q, *J* = 35.4 Hz), 137.0, 129.7, 121.1 (q, *J* = 274.7 Hz), 120.0 (q, *J* = 2.6 Hz), 114.3, 86.2, 42.2, 27.7 (3C); **^{19}F NMR** (565 MHz, Chloroform-*d*) δ -68.1 ppm. **HRMS** (*m/z*) $[M + H]^+$ calculated for $C_{13}H_{14}F_3N_2O_2$ 287.1007; found 287.0984.

2-(6-(Trifluoromethyl)pyridin-3-yl)acetonitrile (9):

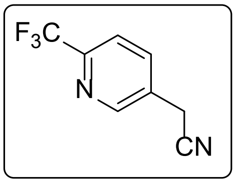

To a solution of cyanoester **15** (5.0 g, 17.47 mmol, 1.0 eq.) in DMSO (50 mL), solution of NaCl (5.10 g, 87.33 mmol, 5.0 eq.) in water (25 mL) was added at room temperature. The suspension was stirred for 16 hours at 100 °C in an unsealed round bottom flask. The reaction mixture was diluted water (200 ml) and the aqueous phase was extracted with EtOAc (3 X 100 mL). The organic layer was washed with brine (100 mL), dried over Na_2SO_4 and solvent was removed under reduced pressure. The crude residue was purified by silica gel column chromatography (20% to 50% EtOAc in hexanes) to give the titled product **9** as a brown solid (2.57 g, 79% yield).

^{1}H NMR (600 MHz, Chloroform-*d*) δ 8.67 (d, *J* = 2.5 Hz, 1H), 7.90 (dd, *J* = 8.2, 2.3 Hz, 1H), 7.72 (d, *J* = 8.1 Hz, 1H), 3.85 (s, 2H); **^{13}C NMR** (150 MHz, Chloroform-*d*) δ 149.3, 148.1 (q, *J* = 35.1 Hz), 137.0, 129.5, 121.3 (q, *J* = 274.1 Hz), 120.8 (q, *J* = 2.8 Hz), 116.0, 21.2; **^{19}F NMR** (565 MHz, Chloroform-*d*) δ -68.1 ppm.

Data matched with those previously reported.[4]

(*E*)-*N*-(3-(Dimethylamino)-2-(4-(methylsulfonyl)phenyl)allylidene)-*N*-methylmethanaminium hexafluorophosphate (8):

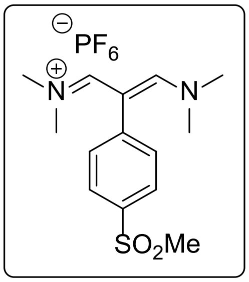

To a solution of 2-(4-(methylsulfonyl)phenyl)acetic acid **7** (10.0 g, 46.68 mmol, 1.0 eq.) in DMF (100 mL), $POCl_3$ (13.77 g, 98.03 mmol, 2.1 eq.) was added dropwise. The reaction mixture was stirred for 3 hours at 80 °C. The reaction mixture was cooled to room temperature. In another 250 mL round bottom flask, a solution of $NaPF_6$ was prepared by addition of HPF_6 (55 wt% in water; 13.63 g, 51.35 mmol, 1.1 eq.) and 5N NaOH solution (10 mL) in water (50 mL). To this solution, above reaction mixture and 5N NaOH solution (35 mL) were added concurrently over 1 hour at 0 °C. The mixture was stirred at the same temperature for 3 hours and solids were collected by filtration. The filtered cake was washed with ice cold water (3 X 50 mL), dried under vacuum to provide desired vinamidinium salt **8** as a yellow solid (18.95 g, 95% yield).

^{1}H NMR (600 MHz, Acetonitrile-*d*$_3$) δ 7.97 (d, J = 8.2 Hz, 2H), 7.56 (d, J = 8.0 Hz, 2H), 7.43 (s, 2H), 3.26 (s, 6H), 3.12 (s, 3H), 2.45 (s, 6H); **^{13}C NMR** (150 MHz, Acetonitrile-*d*$_3$) δ 163.9, 142.2, 139.6, 134.4, 128.2 (4C), 104.6, 49.8 (2C), 44.6 (2C), 40.9; **^{19}F NMR** (565 MHz, Acetonitrile-*d*$_3$) δ -72.2, -73.4 ppm. **HRMS** (*m/z*) $[M]^+$ calculated for $C_{14}H_{21}N_2O_2S$ 281.1324; found 281.1310.

(2*Z*,4*Z*)-5-(Dimethylamino)-4-(4-(methylsulfonyl)phenyl)-2-(6-(trifluoromethyl)pyridin-3-yl)penta-2,4-dienenitrile (16):

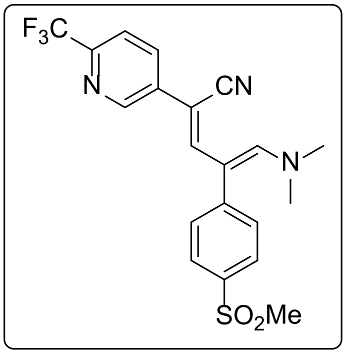

To a solution of pyridine derivative **9** (1.1 g, 5.91 mmol, 1.0 eq.) and vinamidinium salt **8** (3.02 g, 7.09 mmol, 1.2 eq.) in THF (15 mL), a solution of KO^{t}Bu (1.46 g, 13.00 mmol, 2.2 eq.) in THF (5 mL) was added dropwise over 10 minutes at 0 °C. The reaction mixture was stirred and allowed to warm from 0 °C to room temperature over 2 hours. The reaction mixture was diluted with water (50 ml) and product was extracted by EtOAc (3 X 50 mL). The organic layer was dried over Na_2SO_4 and solvent was removed under reduced pressure. The crude product was purified by silica gel column chromatography (30% to 70% EtOAc in hexanes) to afford the titled product **16** as an orange solid (2.22 g, 89% yield).

^{1}H NMR (600 MHz, DMSO-*d*$_6$) δ 8.79 (d, J = 2.4 Hz, 1H), 7.94 (d, J = 7.9 Hz, 1H), 7.91 (d, J = 7.9 Hz, 2H), 7.85 (s, 1H), 7.82 (d, J = 8.4 Hz, 1H), 7.55 (s, 1H), 7.52 (d, J = 8.0 Hz, 2H), 3.18 (s, 3H), 2.82 (s, 6H); **^{13}C NMR** (150 MHz, DMSO-*d*$_6$) δ 155.0, 150.6, 145.3, 142.8 (q, J = 33.8, 33.1 Hz), 142.2, 140.1, 137.2, 133.3, 132.0, 126.9 (4C), 122.4 (q, J = 273.1 Hz), 121.1 (d, J = 2.9 Hz), 117.8, 108.7, 44.4 (2C), 40.5; **^{19}F NMR** (565 MHz, DMSO-*d*$_6$) δ -65.8, -69.5, -70.8 ppm. **HRMS** (*m/z*) $[M + H]^+$ calculated for $C_{20}H_{19}F_3N_3O_2S$ 422.1150; found 422.1130.

MMV 390048 (1):

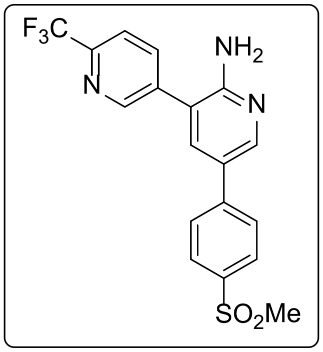

A suspension of nitrile derivative **16** (2.2 g, 5.22 mmol, 1.0 eq.) in NH_4OH (28%; 15 mL, 104.4 mmol, 20.0 eq.) was heated at 100 °C for 16 hours in a sealed glass reactor. The reaction mixture was cooled to 10 °C and solids were collected by filtration. The filtered cake was washed with water (20 mL), MTBE (2 X 20 mL) and dried under vacuum to yield the final product MMV048 **1** as an orange solid (1.71 g, 83% yield, 96.0% purity by qNMR). The API was recrystallized from hot acetonitrile to get 99.42% purity by qNMR (100 A% by HPLC)

^{1}H NMR (600 MHz, DMSO-*d*6) *δ* 8.93 (s, 1H), 8.52 (s, 1H), 8.26 (d, *J* = 7.9 Hz, 1H), 8.01 – 7.94 (m, 5H), 7.91 (s, 1H), 6.36 (s, 2H), 3.25 (s, 3H); **^{13}C NMR** (150 MHz, DMSO-*d*6) *δ* 157.5, 150.6, 147.7, 145.6 (q, *J* = 33.7 Hz), 143.0, 139.0, 138.9, 137.7, 137.4, 128.1 (2C), 126.6 (2C), 123.6, 122.3 (q, *J* = 273.8 Hz), 121.3 (q, *J* = 2.9 Hz), 116.3, 44.1; **^{19}F NMR** (565 MHz, DMSO-*d*6) *δ* -66.2 ppm. **HRMS** (*m/z*) $[M + H]^+$ calculated for $C_{18}H_{13}F_3N_3O_2S$ 394.0837; found 394.0816.

Data matched with those previously reported.[5]

References:

1. Aikawa, K.; Nakamura, Y.; Yokota, Y.; Toya, W. and Mikami, K. *Chem. Eur. J.* **2015**, *21*, 96 – 100.
2. Davies, I. W.; Marcoux, J.-F. and Taylor, J. *Org. Synth*. **2003**, *80*, 200 – 206.
3. Ross, R.; Renga, J. M.; Bland, D. C.; Roth, G.; Fung, A. P. and Davis, C. S. WO 2013/148338 Al, 3 October **2013**.
4. Stazi, F.; Maton, W.; Castoldi, D.; Westerduin, P.; Curcuruto, O. and Bacchi, S. *Synthesis* **2010**, *19*, 3332 – 3338.
5. Younis, Y.; Douelle, F.; Feng, T.-S.; Cabrera, D. G.; Manach, C. L.; Nchinda, A. T.; Duffy, S.; White, K. L.; Shackleford, D. M.; Morizzi, J.; Mannila, J.; Katneni, K.; Bhamidipati, R.; Zabiulla, K. M.; Joseph, J. T.; Bashyam, S.; Waterson, D.; Witty, M. J.; Hardick, D.; Wittlin, S.; Avery, V.; Charman, S. A.; Chibale, K. *J. Med. Chem*. **2012**, *55*, 3479 – 3487.

NMR spectra:

^{1}H NMR Spectra of pyridine derivative 13 in $CDCl_3$ at 600 MHz:

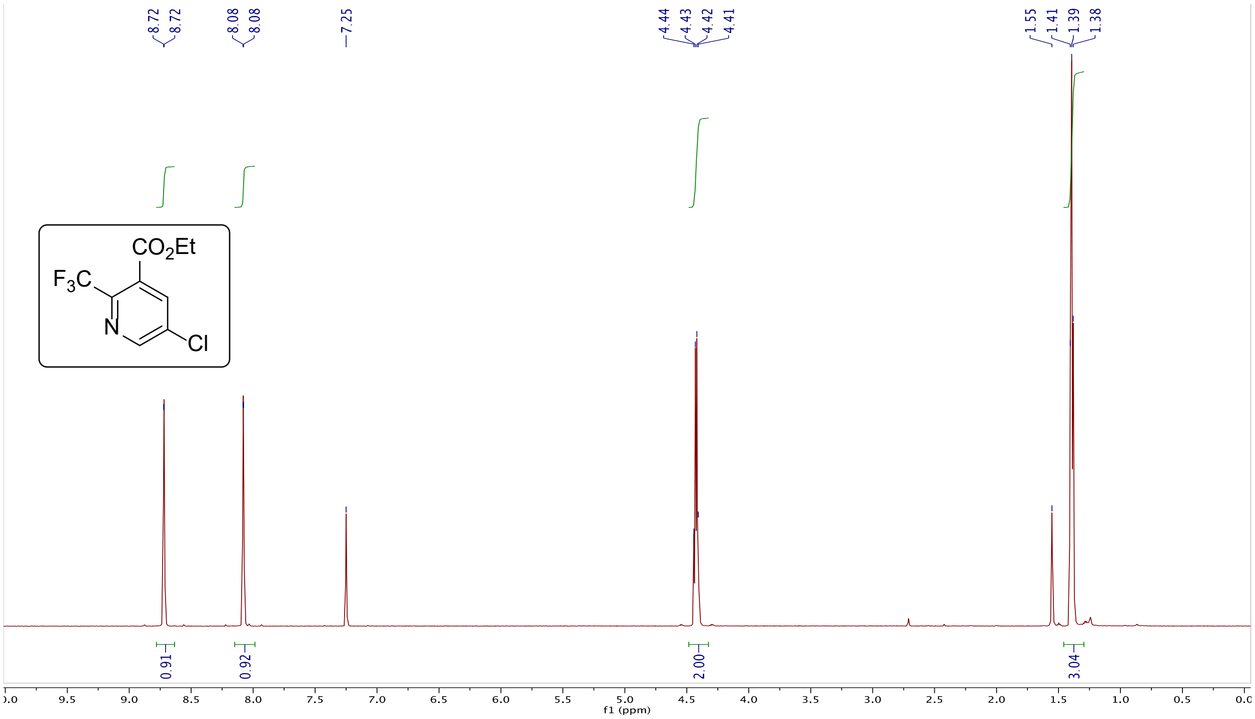

^{13}C NMR Spectra of pyridine derivative 13 in $CDCl_3$ at 150 MHz:

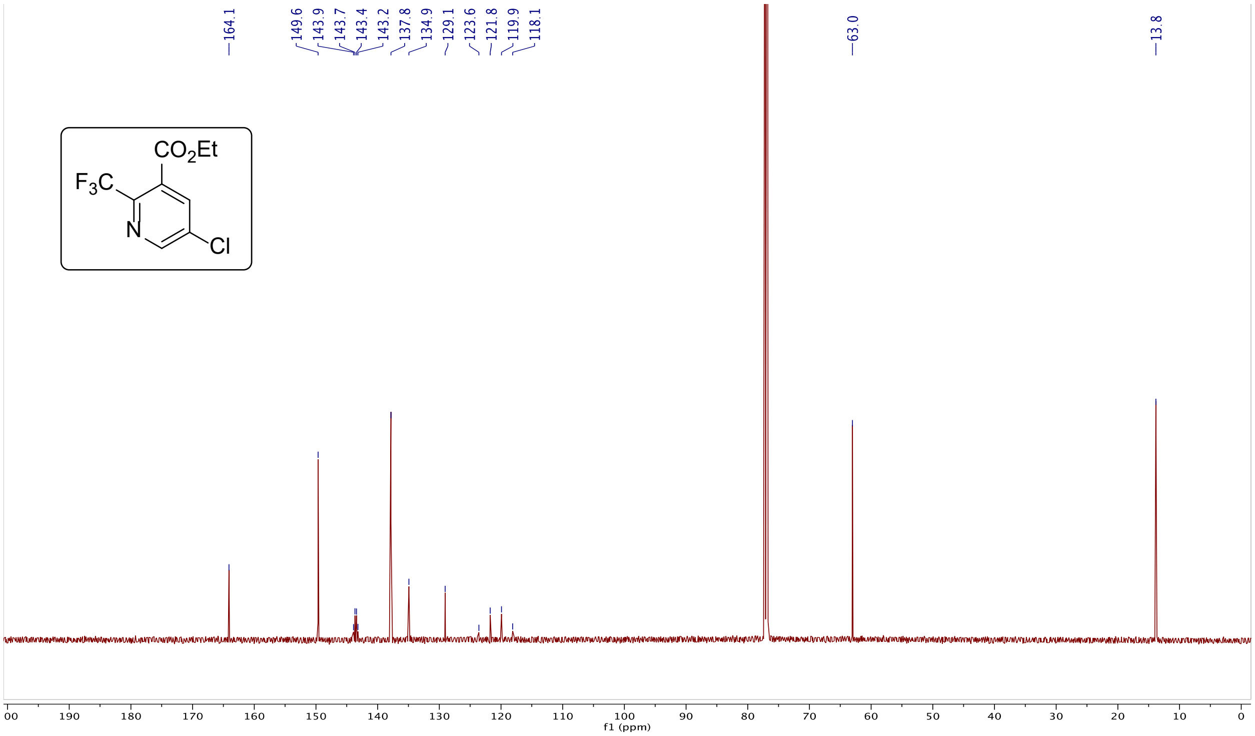

^{19}F NMR Spectra of pyridine derivative 13 in $CDCl_3$ at 565 MHz:

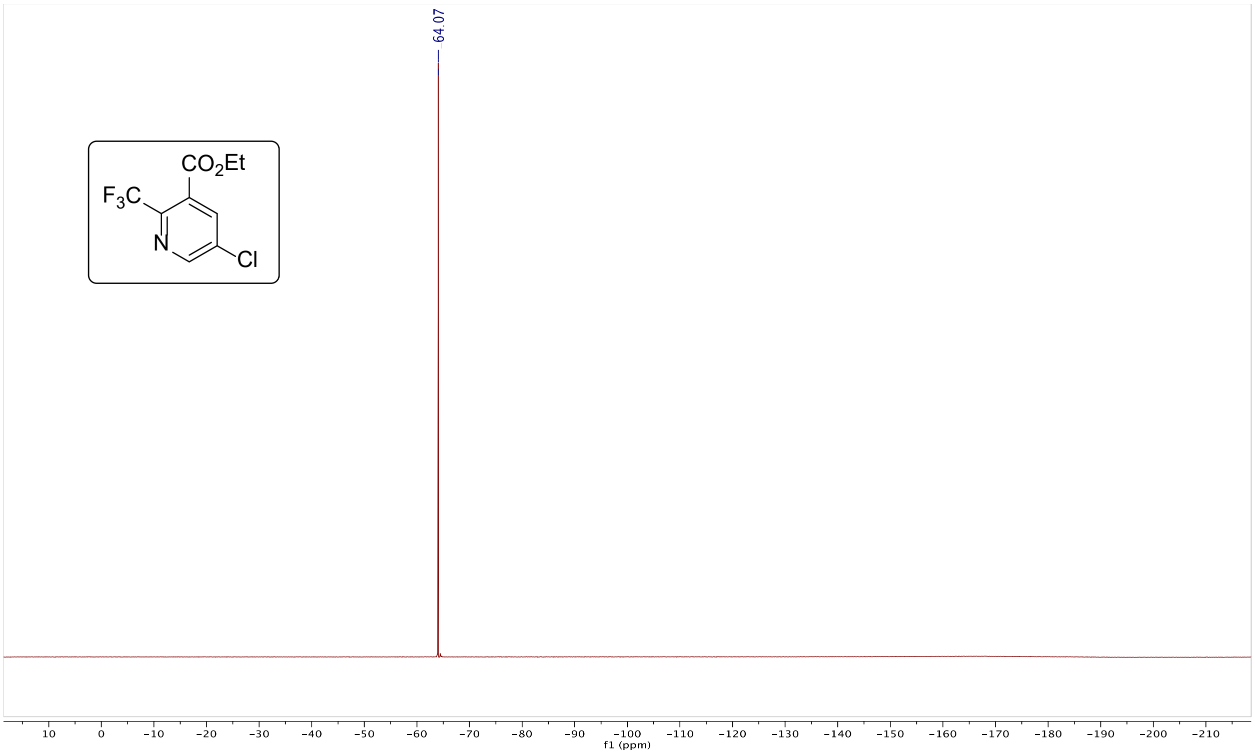

^{1}H NMR Spectra of 5-chloro-2-trifluoropyridine 14 in $CDCl_3$ at 600 MHz:

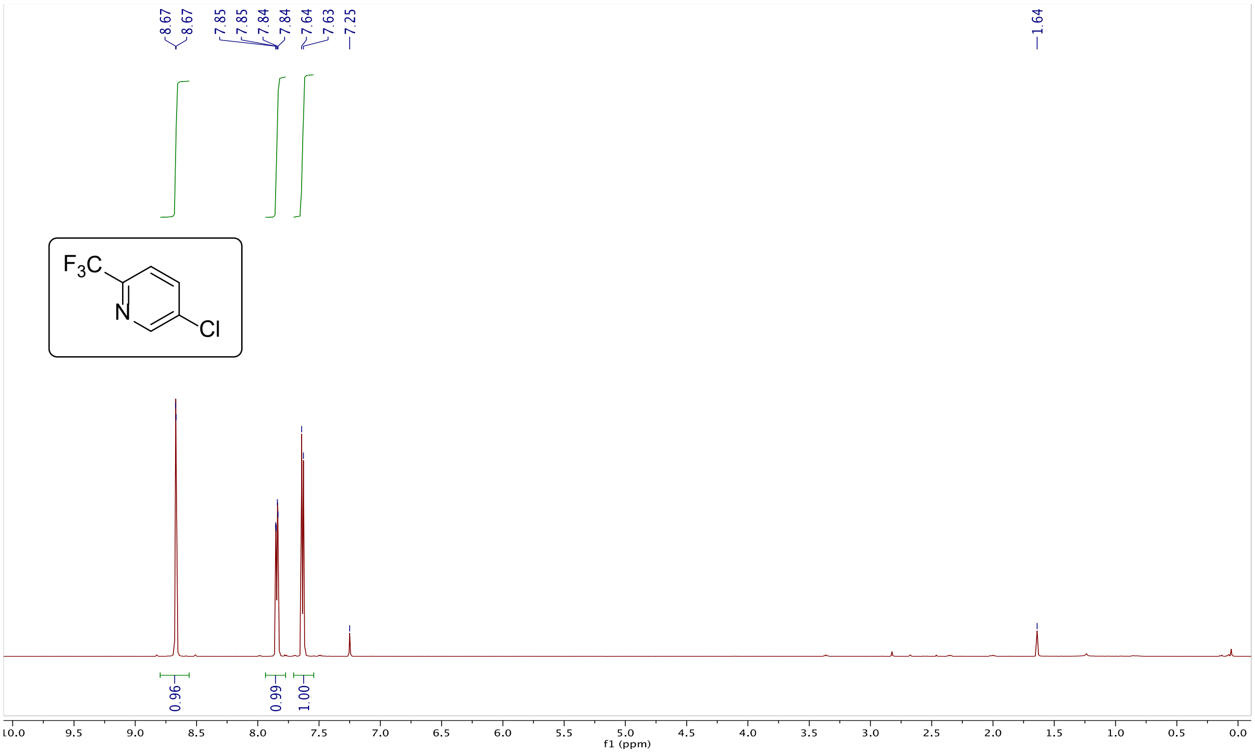

^{13}C NMR Spectra of 5-chloro-2-trifluoropyridine 14 in $CDCl_3$ at 150 MHz:

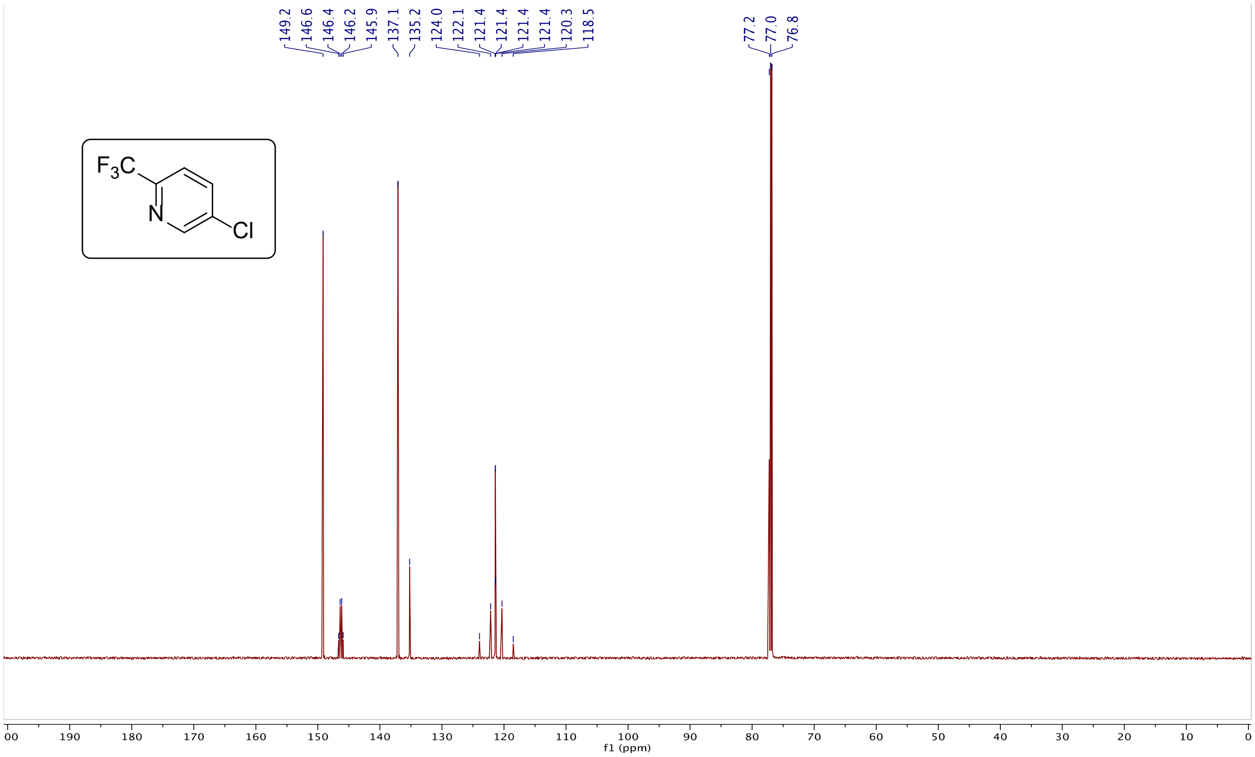

^{19}F NMR Spectra of 5-chloro-2-trifluoropyridine 14 in $CDCl_3$ at 565 MHz:

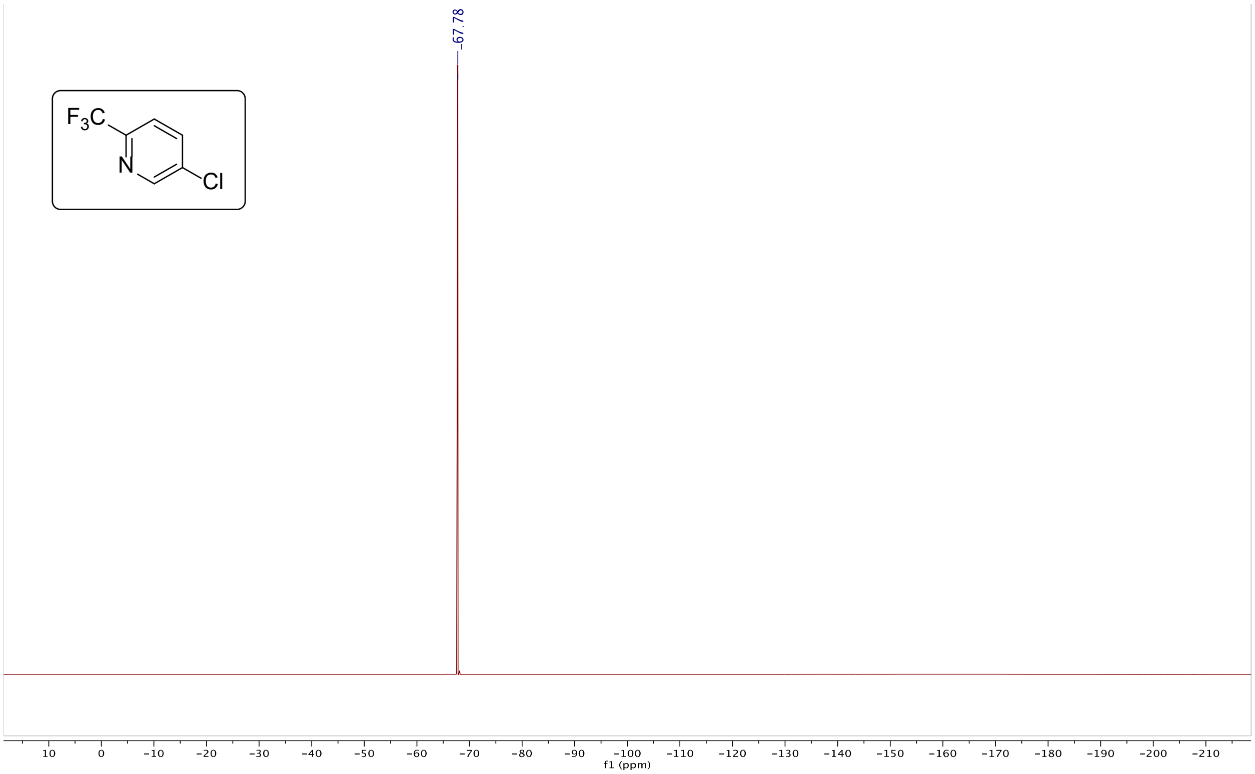

^{1}H NMR Spectra of cyanoester 15 in $CDCl_3$ at 600 MHz:

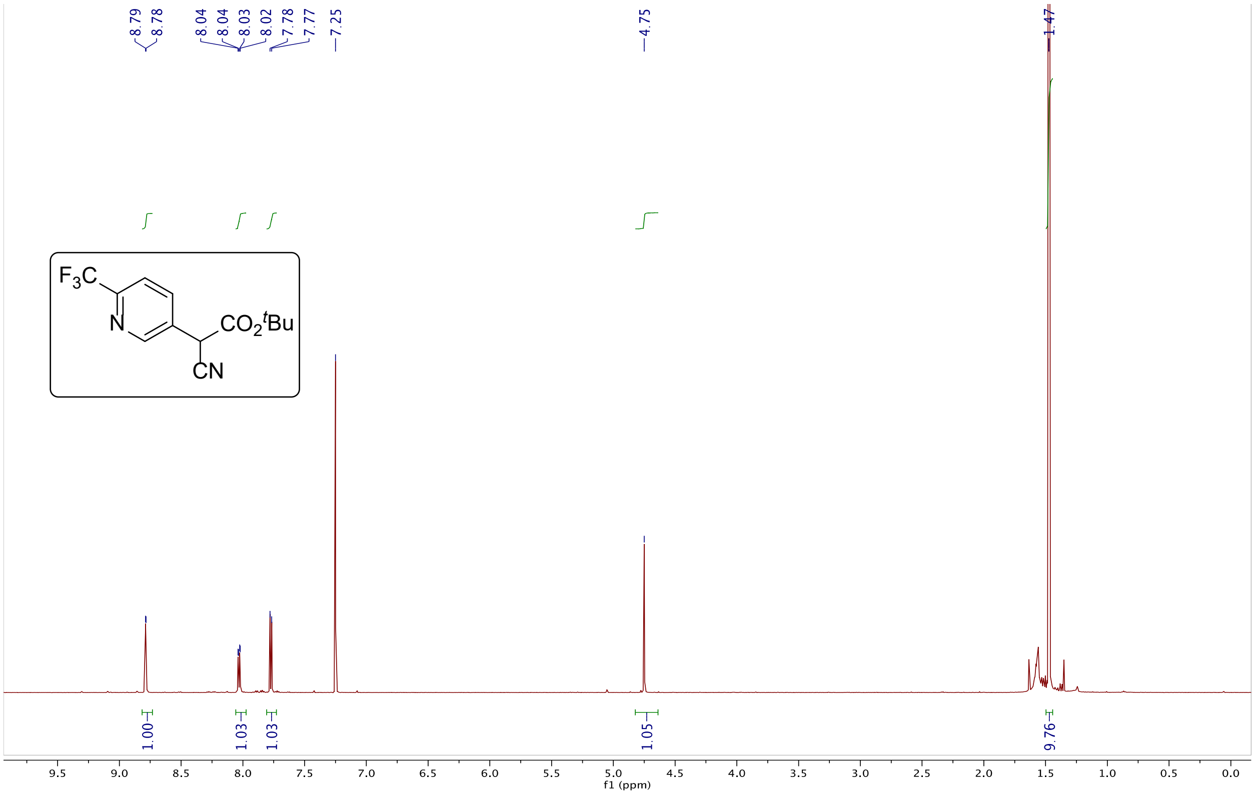

^{13}C NMR Spectra of cyanoester 15 in $CDCl_3$ at 150 MHz:

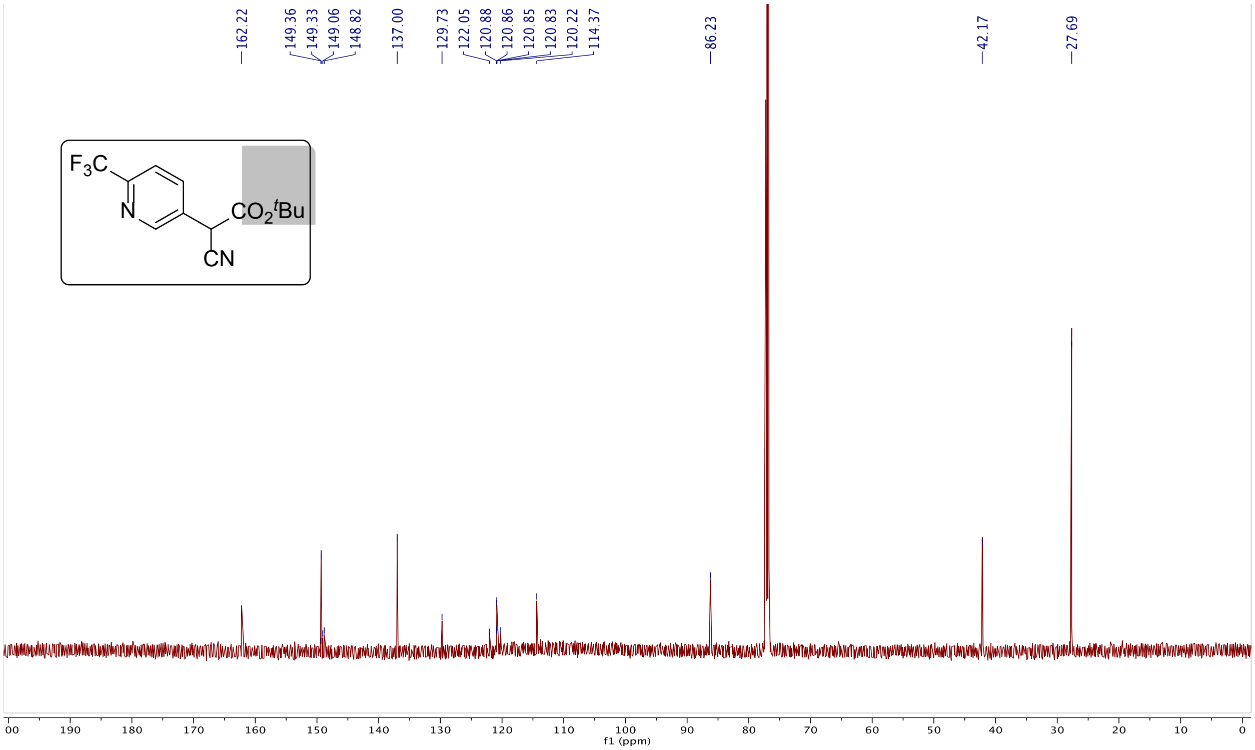

^{19}F NMR Spectra of cyanoester 15 in $CDCl_3$ at 565 MHz:

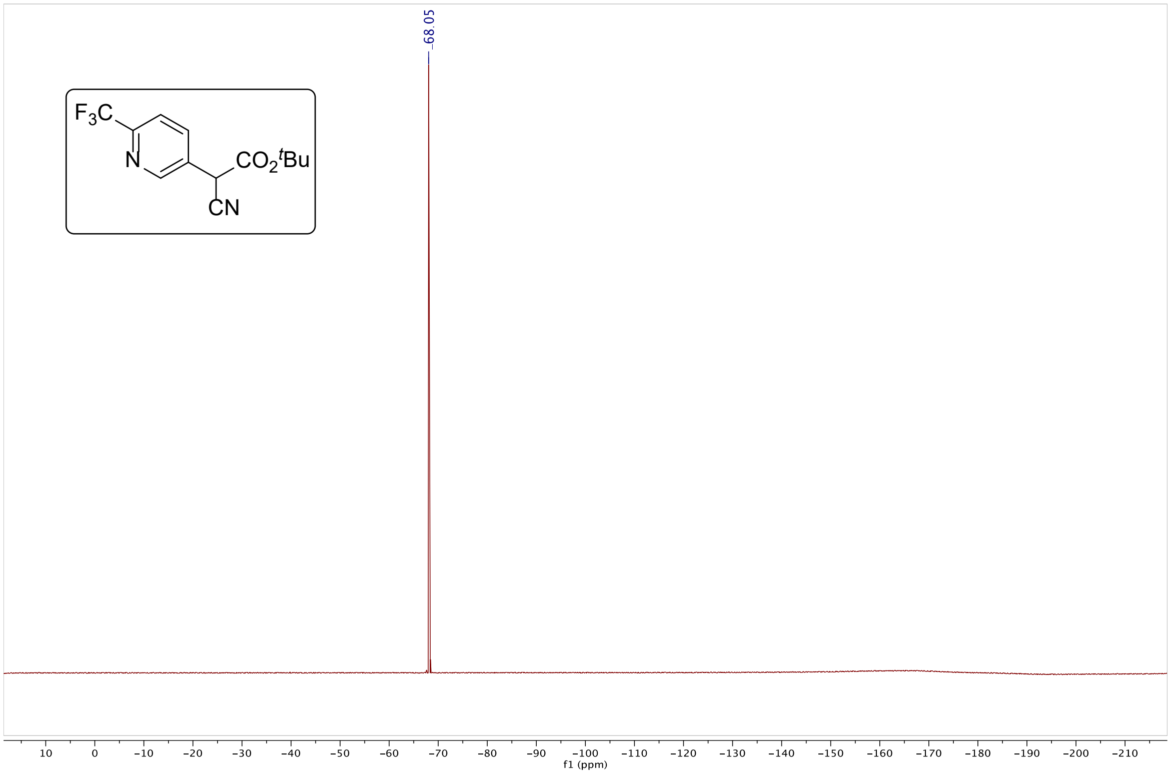

^{1}H NMR Spectra of nitrile derivative 9 in $CDCl_3$ at 600 MHz:

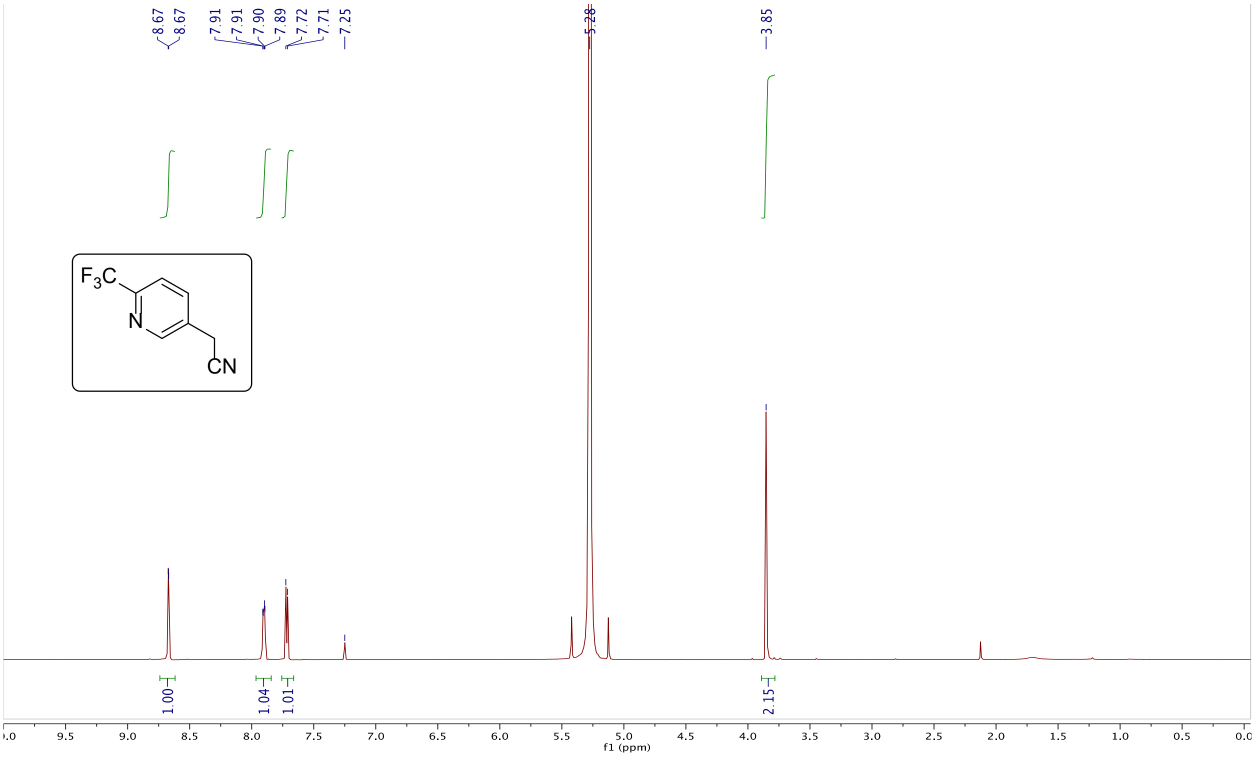

^{13}C NMR Spectra of nitrile derivative 9 in $CDCl_3$ at 150 MHz:

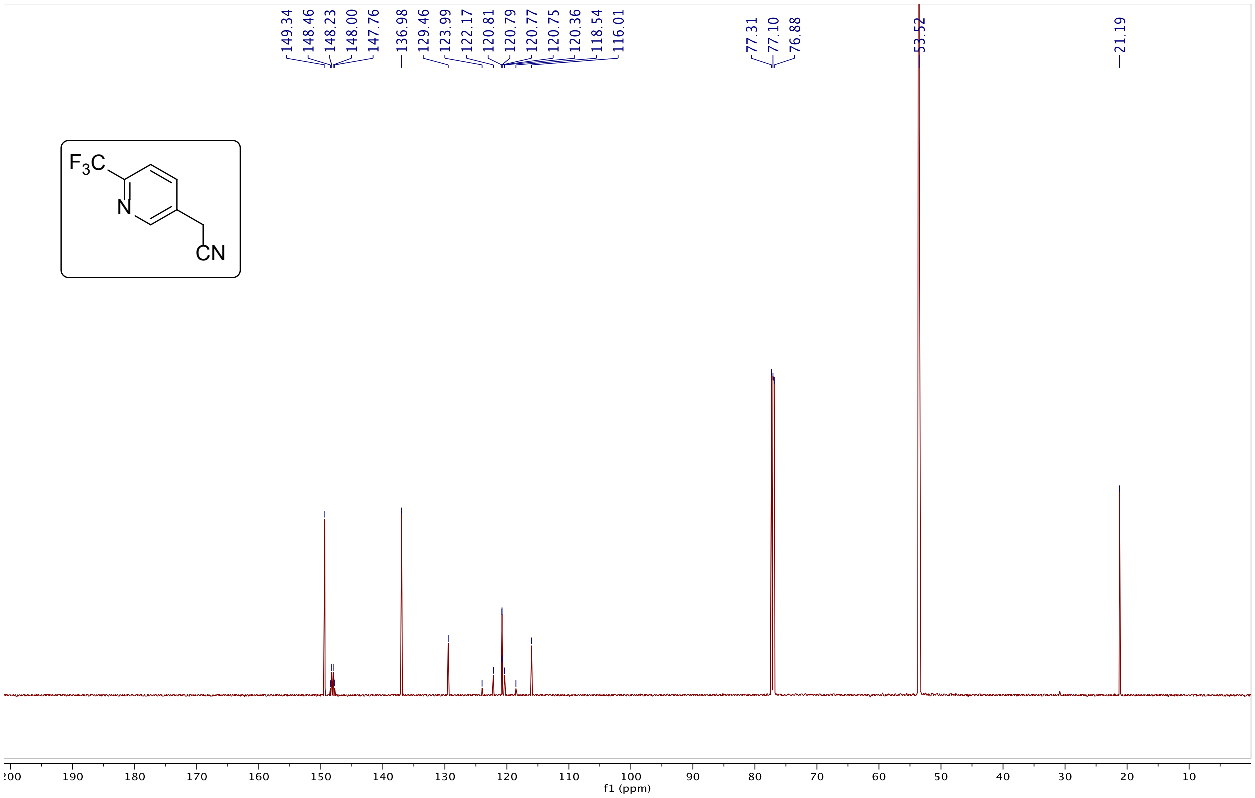

^{19}F NMR Spectra of nitrile derivative 9 in $CDCl_3$ at 565 MHz:

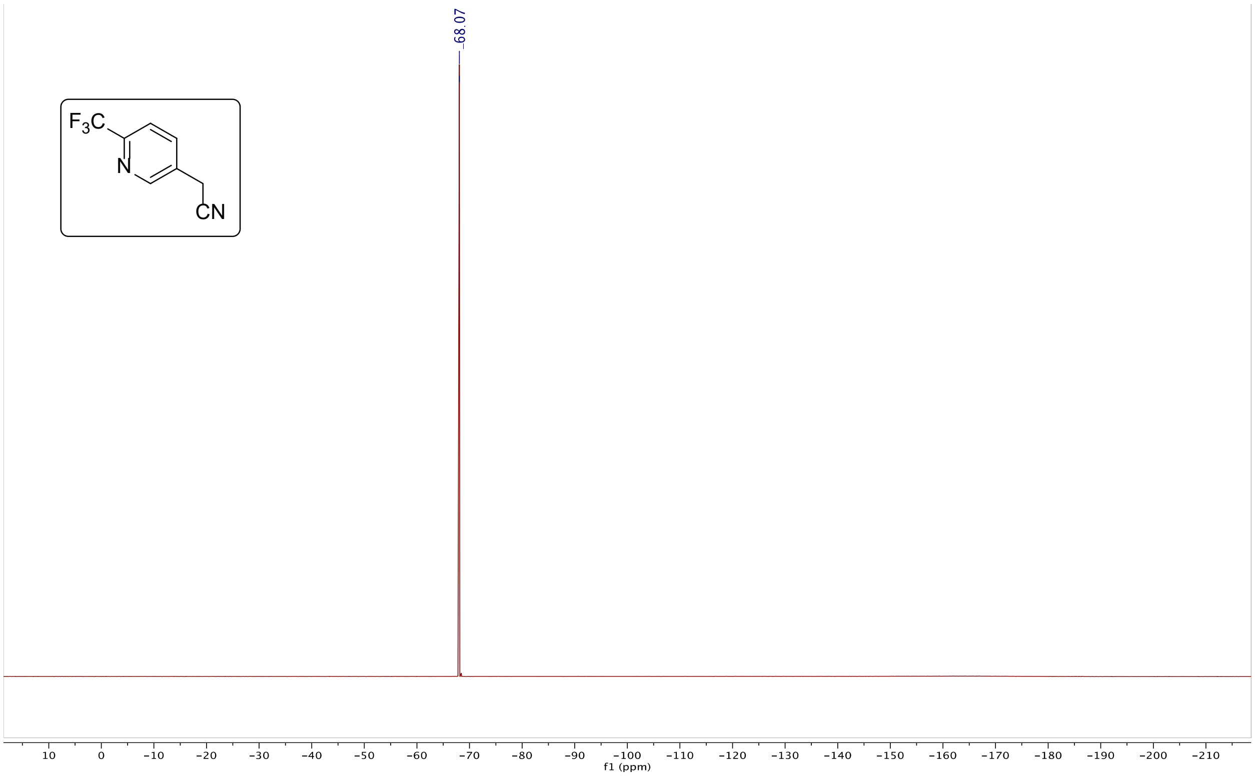

^{1}H NMR Spectra of vinamidinium salt 8 in CD_3CN at 600 MHz:

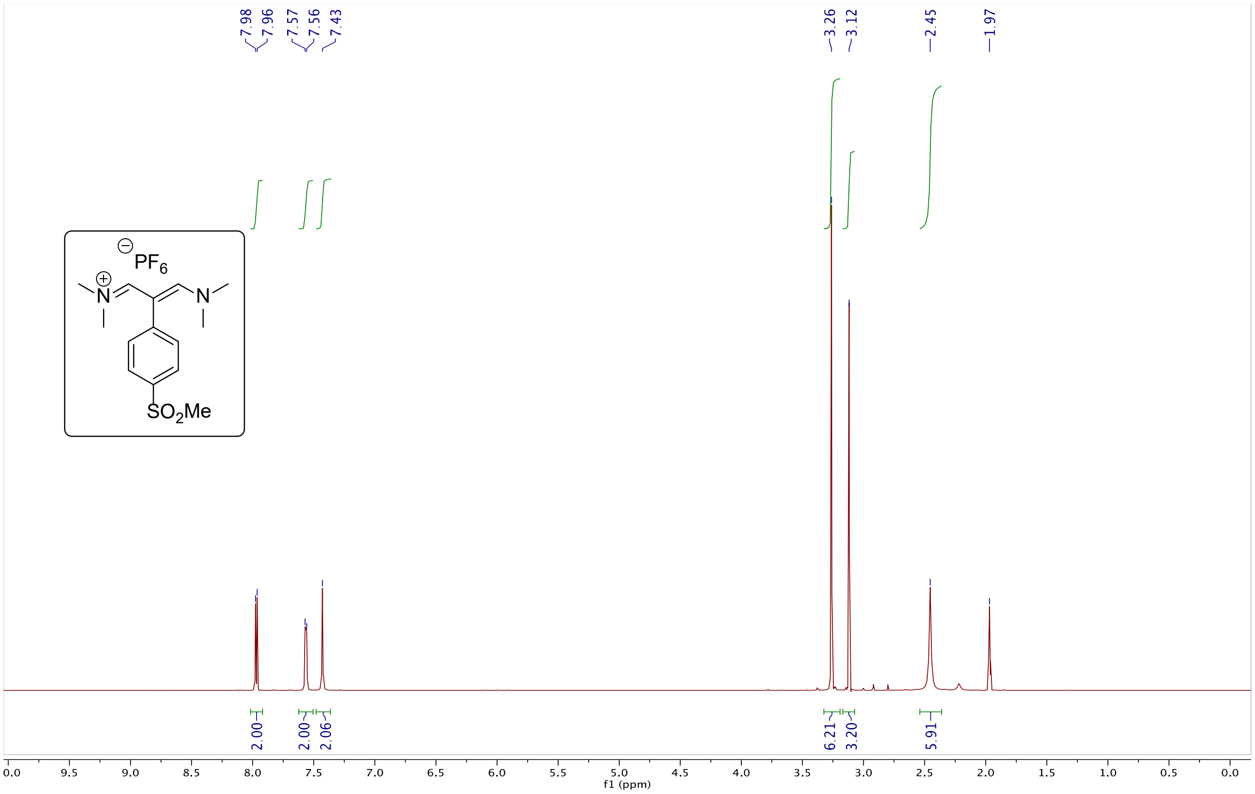

^{13}C NMR Spectra of vinamidinium salt 8 in CD_3CN at 150 MHz:

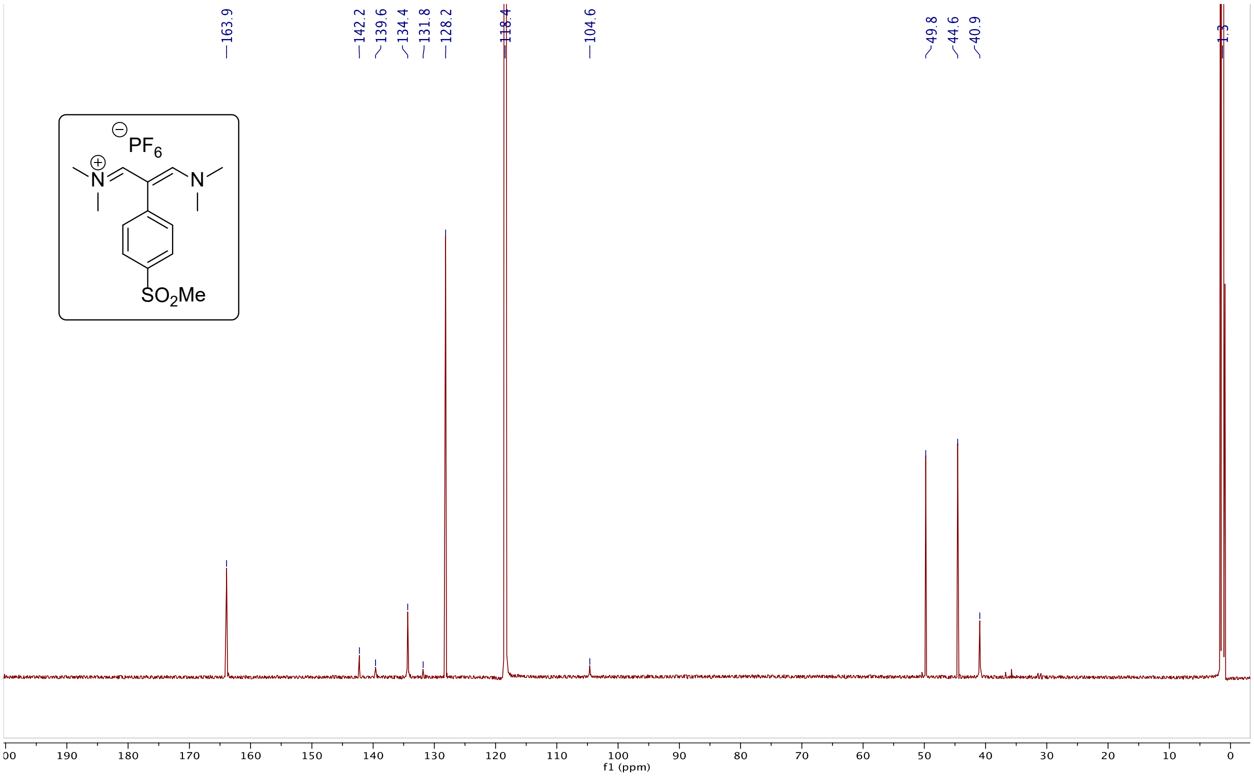

^{19}F NMR Spectra of vinamidinium salt 8 in CD_3CN at 565 MHz:

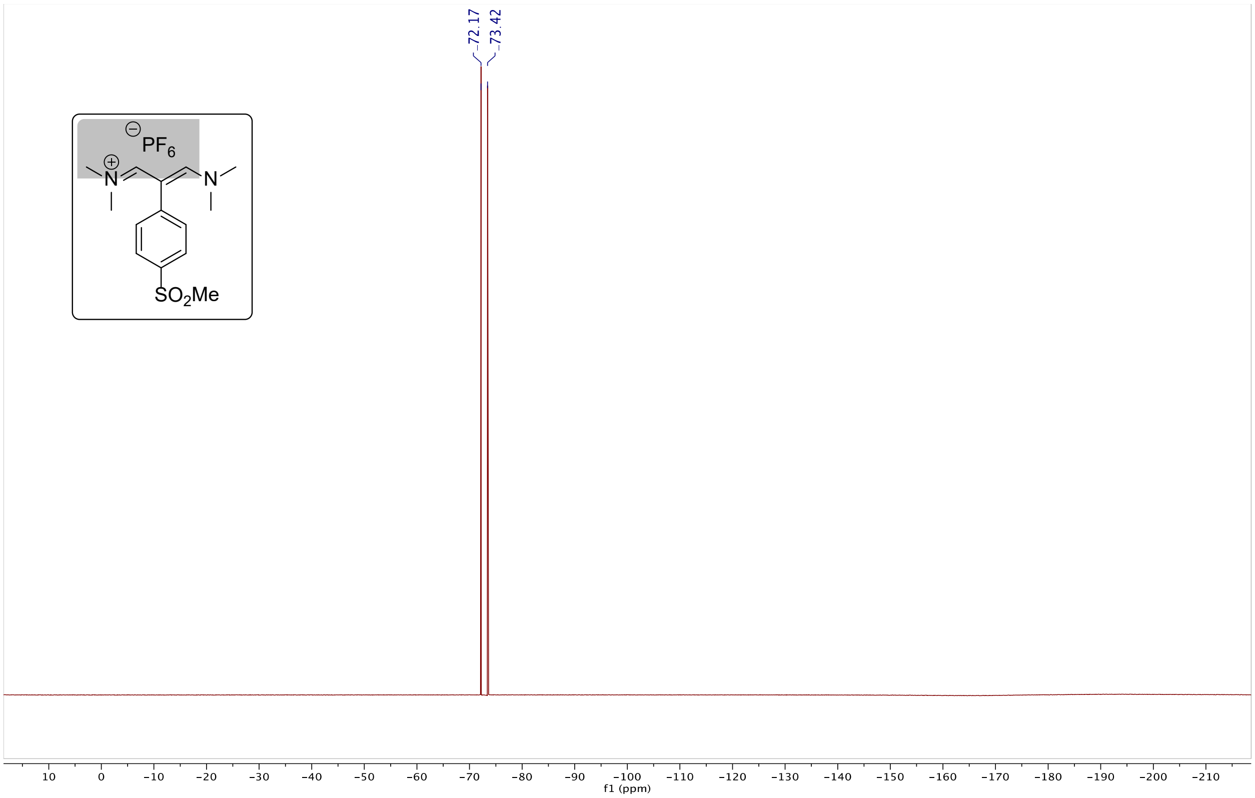

^{1}H NMR Spectra of nitrile derivative 16 in DMSO-d_6 at 600 MHz:

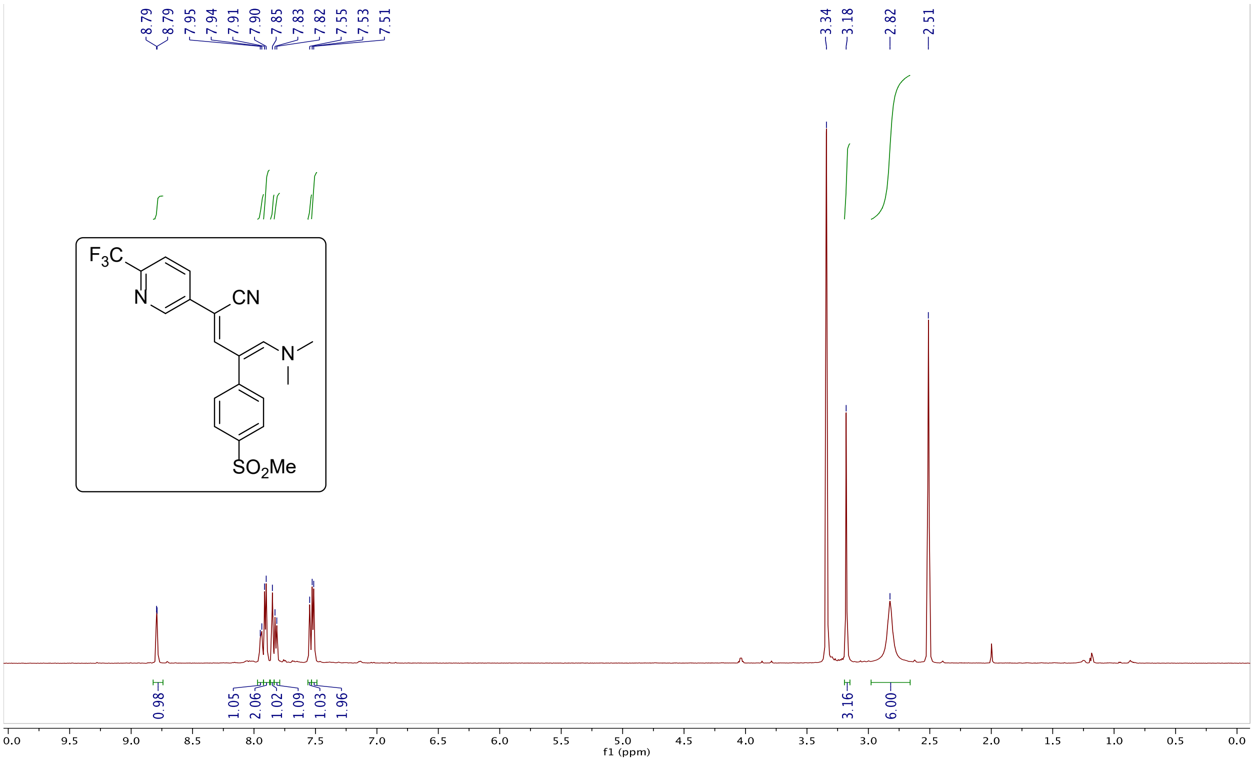

^{13}C NMR Spectra of nitrile derivative 16 in DMSO-d_6 at 150 MHz:

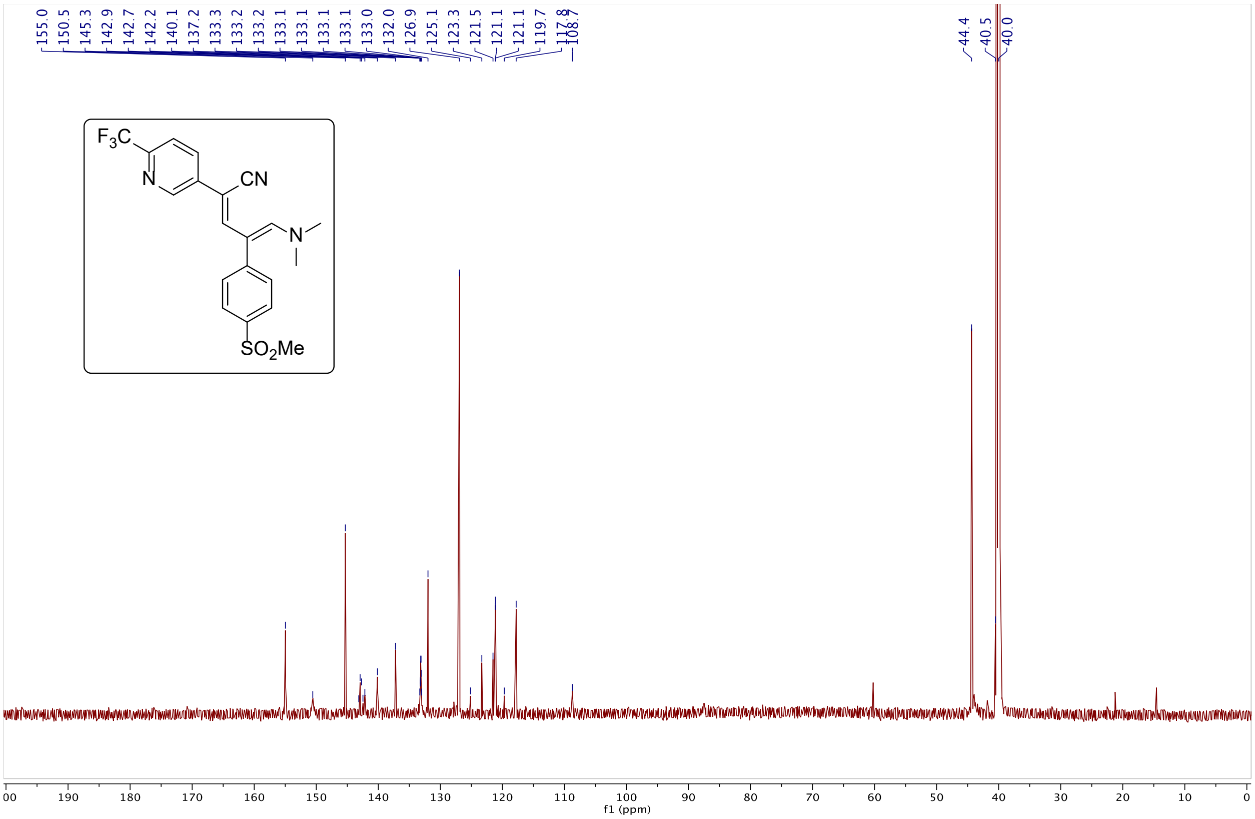

^{19}F NMR Spectra of nitrile derivative 16 in DMSO-d_6 at 565 MHz:

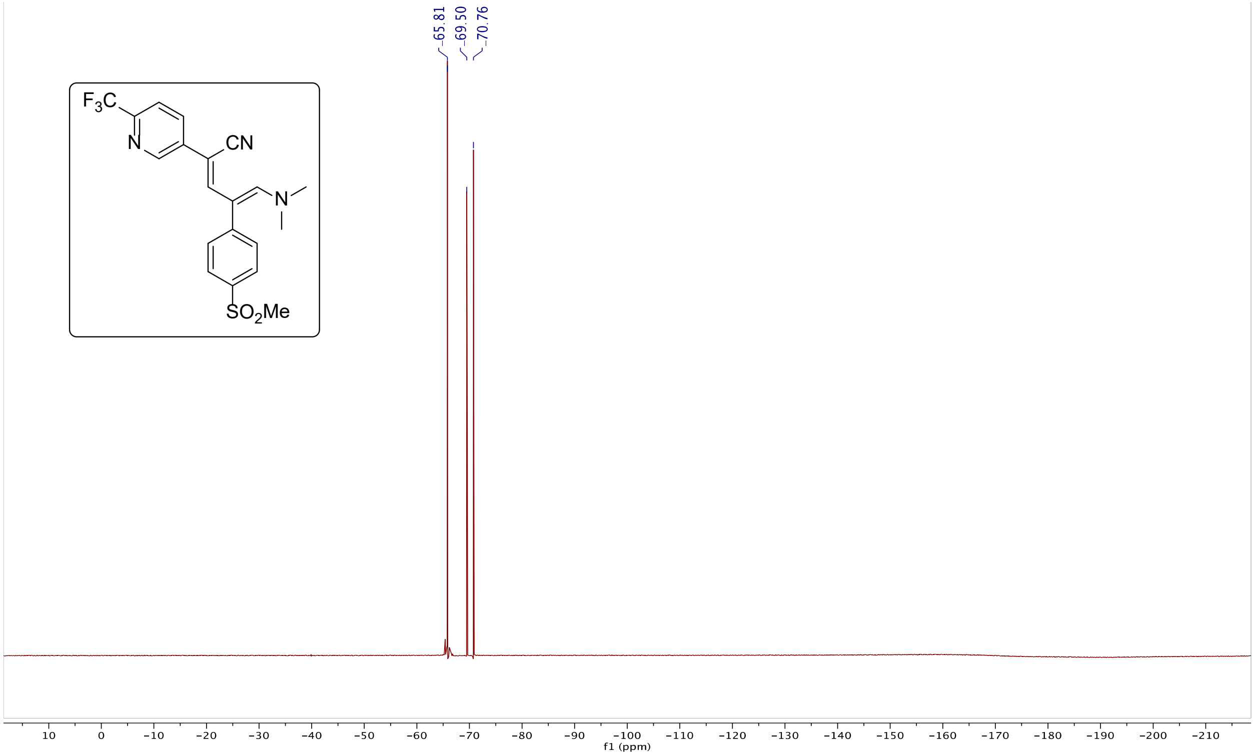

^{1}H NMR Spectra of MMV048 in DMSO-d_6 at 600 MHz:

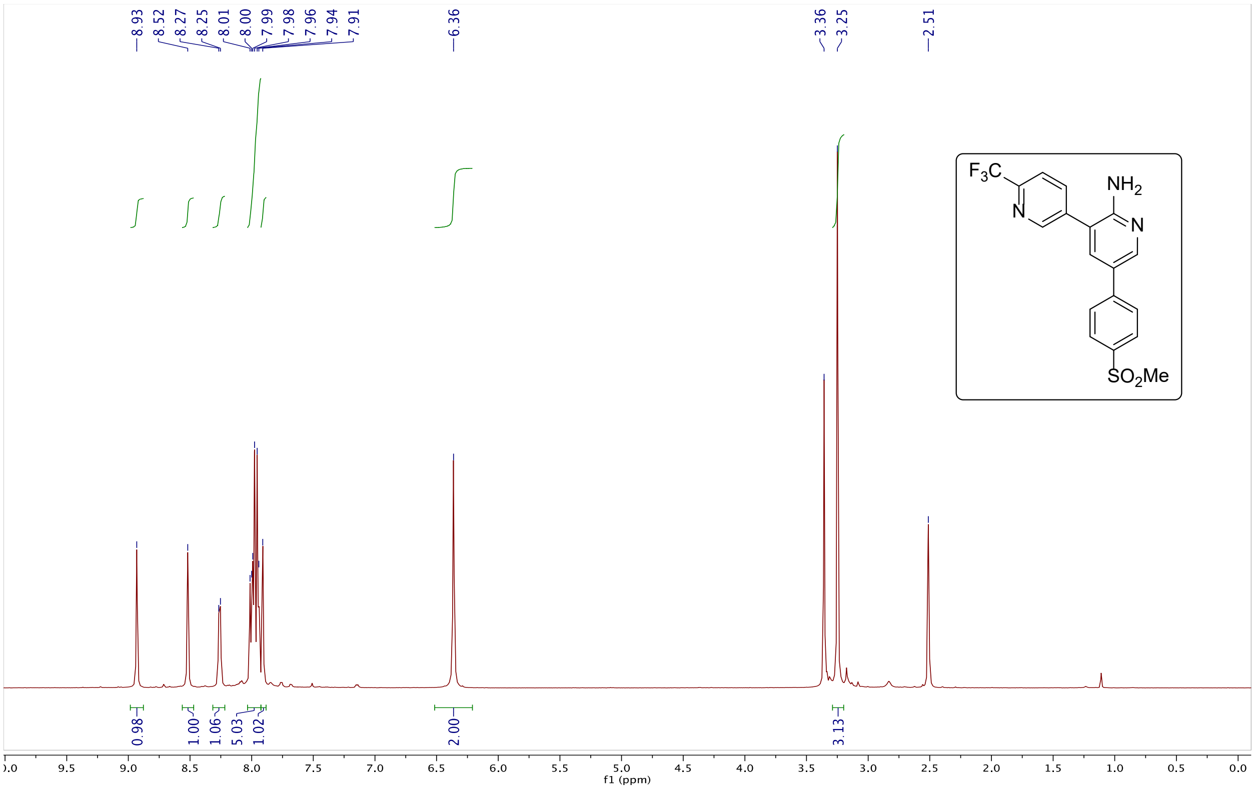

^{13}C NMR Spectra of MMV048 in DMSO-d_6 at 150 MHz:

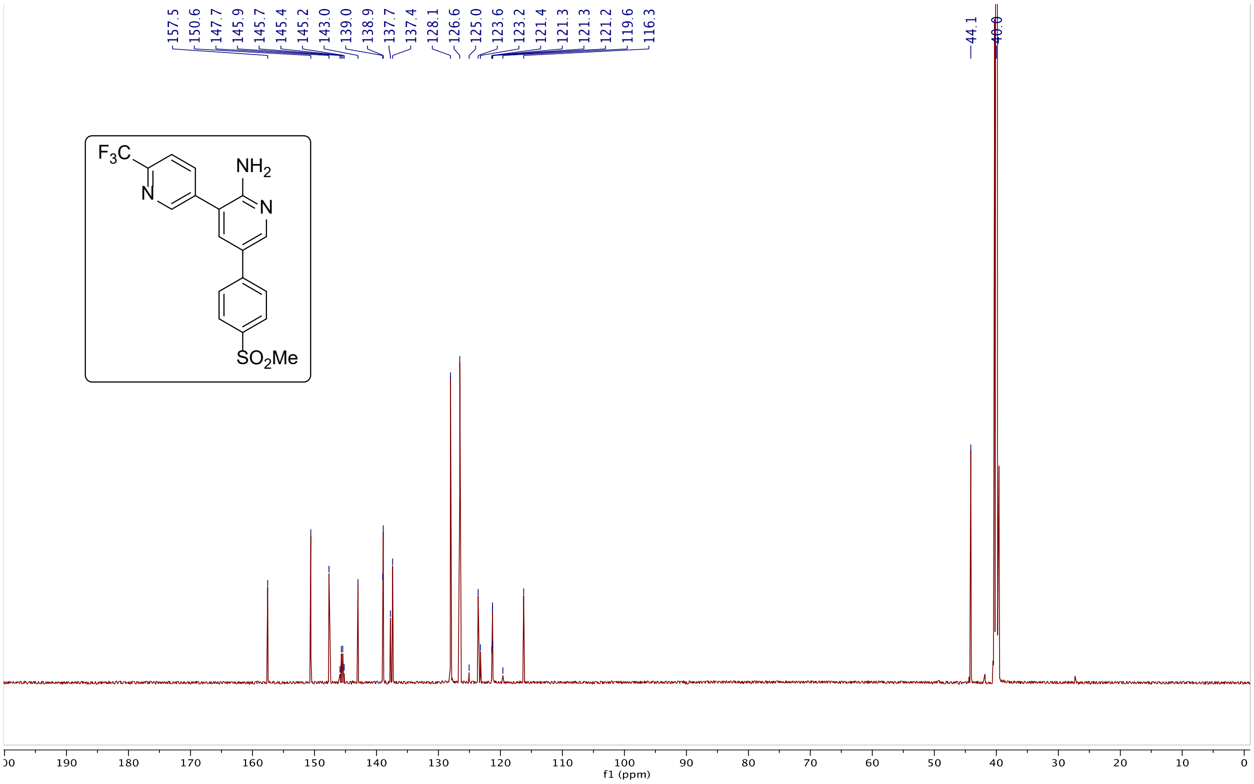

^{19}F NMR Spectra of MMV048 in DMSO-d_6 at 565 MHz:

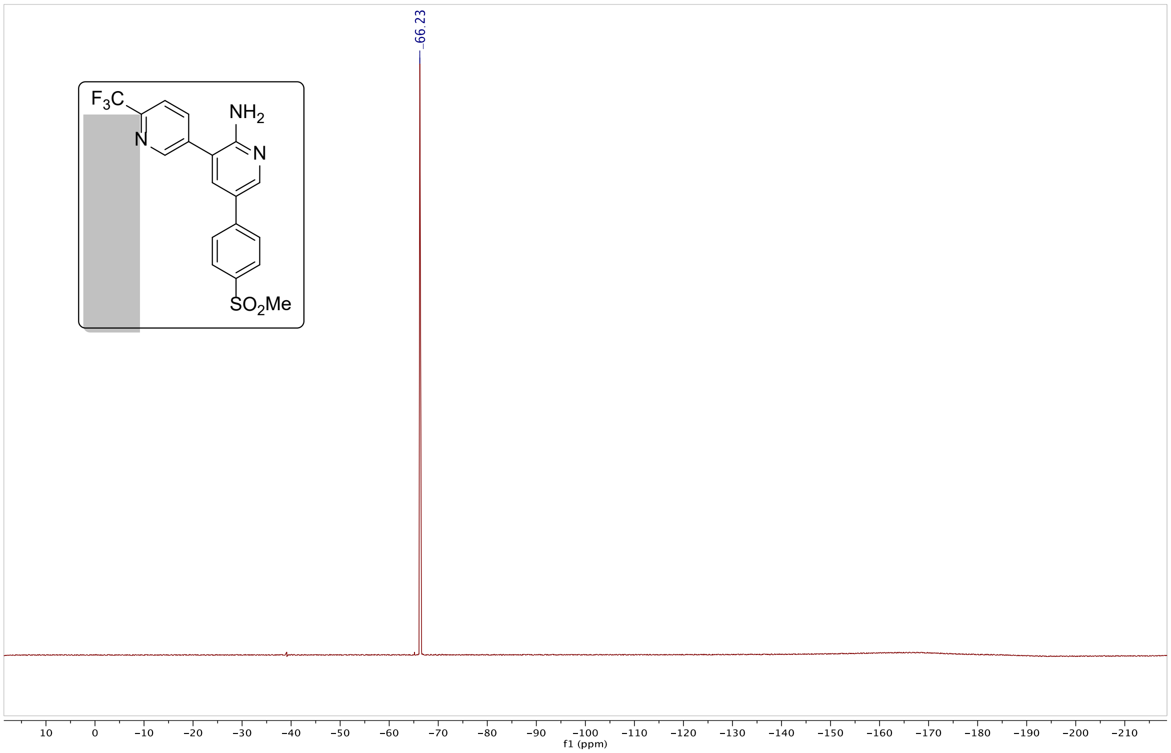

HPLC Chromatogram of MMV0048:

```
=====================================================================
Acq. Operator   : SYSTEM                          Seq. Line :   2
Acq. Instrument : 1260-2                           Location :   2
Injection Date  : 4/19/2021 3:11:21 PM                  Inj :   1
                                                Inj Volume : 1.000 µl
Method          : C:\Chem32\1\Data\Dinesh\EIDD 2021-04-19 15-01-29\TBI_Isocratic_50% ACN.M (
                  Sequence Method)
Last changed    : 4/19/2021 3:01:29 PM by SYSTEM
Method Info     : XDB-C18 4.6 x 250 mm 5-micron; 75% 0.1% H3PO4:25% ACN, 1.5 mL/min, 30C
```

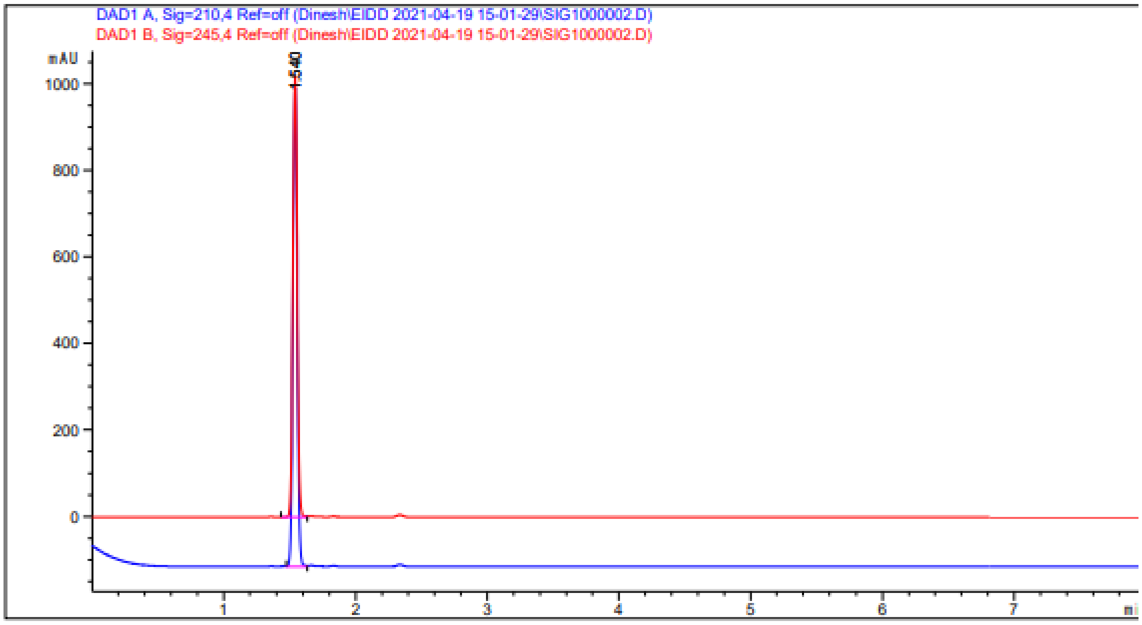


```
=====================================================================
                         Area Percent Report
=====================================================================

Sorted By             :      Signal
Multiplier            :      1.0000
Dilution              :      1.0000
Use Multiplier & Dilution Factor with ISTDs


Signal 1: DAD1 A, Sig=210,4 Ref=off

Peak RetTime Type  Width     Area      Height     Area
  #   [min]        [min]   [mAU*s]     [mAU]        %
----|-------|----|-------|----------|----------|--------|
   1   1.540 VV    0.0358 2540.52344 1107.04578 100.0000

Totals :                  2540.52344 1107.04578
```

Signal 2: DAD1 B, Sig=245,4 Ref=off

Peak #	RetTime [min]	Type	Width [min]	Area [mAU*s]	Height [mAU]	Area %
1	1.540	VV	0.0357	2338.25562	1021.92767	100.0000

Totals : 2338.25562 1021.92767

*** End of Report ***
